# Crossroads in the Learning Brain: The Neural Overlap Between Arithmetic and Phonological Processing

**DOI:** 10.1002/hbm.70446

**Published:** 2026-01-13

**Authors:** Aymee Alvarez‐Rivero, Lien Peters, Marc F. Joanisse, Nadine Gaab, Daniel Ansari

**Affiliations:** ^1^ The University of Western Ontario London Ontario Canada; ^2^ University of Ghent Ghent Belgium; ^3^ Harvard Graduate School of Education Cambridge Massachusetts USA

**Keywords:** arithmetic, conjunction analysis, fMRI, phonological processing, representational similarity analysis

## Abstract

Robust behavioral evidence suggests an association between reading and math performance. Moreover, previous neuroimaging evidence suggests that arithmetic fact retrieval is supported by similar areas along the perisylvian language network as those typically involved in phonological processing. However, the neural correlates of these abilities have been mostly studied in isolation, and therefore remains unclear whether these abilities recruit functionally overlapping brain areas. We addressed this question by using functional magnetic resonance imaging to measure brain activity during an arithmetic and a word rhyming task. We then used both a test of univariate overlap and a rigorous pattern similarity analysis to provide a more nuanced assessment of brain‐level associations across both domains. We identified clusters of significant overlap along the left inferior frontal gyrus, the left inferior temporal gyrus, and the right posterior cerebellum in adults; as well as multiple clusters along the left frontal gyrus in children. Moreover, we found significant similarity between the patterns corresponding to both abilities along the clusters of overlap. However, contrary to our expectations, we observed higher similarity between phonological processing and large problems than small problems, which grants the need for further research about the role of arithmetic strategies in this relationship. Our findings represent a contribution to the literature examining the potential links between the brain regions supporting arithmetic and word reading by providing direct, within‐participant statistical evidence of the long‐hypothesized overlap between these processes at the neural level.

## Graphical abstract

Robust behavioral evidence suggests an association between reading and math performance; however, the neural correlates of these abilities have been mostly studied in isolation. Our findings provide direct, within‐participant statistical evidence of the long‐hypothesized overlap between phonological processing and arithmetic at the neural level using univariate and multivariate methods.

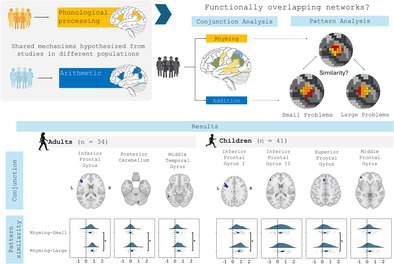

## 1 Introduction

Math and reading abilities have an important influence on individuals' academic success, socio‐economic status and even health outcomes (Brown 2021; McLaughlin et al. 2014; Ritchie and Bates 2013). Moreover, math and reading abilities are strongly correlated, concurrently and longitudinally (Ding and Homer 2020; Ischebeck et al. 2009; Korhonen et al. 2012; Korpershoek et al. 2015; Korpipää et al. 2017; see Singer and Strasser 2017 for a meta‐analysis). The co‐occurrence of Dyslexia—a specific difficulty for reading—and Dyscalculia—a specific difficulty for mathematics—far exceeds what would be expected if the two conditions had independent etiology (Clercq‐Quaegebeur et al. 2018; Dirks et al. 2008; Joyner and Wagner 2020; Landerl and Moll 2010; Peterson et al. 2017; Simmons and Singleton 2008; Willcutt et al. 2019), at least in the context of predominantly Western, educated, industrialized, rich, and democratic (WEIRD) samples (Henrich et al. 2010). These findings suggest the possibility of common risk factors that influence the acquisition of both numeracy and literacy. And yet, most of what we know about the neural substrates that support these abilities comes from research that has studied each of these processes independently. Understanding the common factors related to the cognitive and neural bases of math and reading is therefore critical not only for advancing theoretical models of how these abilities are acquired, but also for informing empirical efforts to foster learning across both domains.

The correlations between different math and reading abilities could be explained, to some extent, by general‐domain factors such as intelligence, executive functions, or processing speed (Geary et al. 2020; Spiegel et al. 2021; Ünal et al. 2023). However, in addition to these broad influences, recent research has suggested that phonological processing abilities—a specific precursor of reading acquisition defined as the ability to perceive, manipulate, and produce the sounds of language—may also uniquely contribute to the development of verbal aspects of mathematics, in particular arithmetic (De Smedt 2018; Pollack and Ashby 2018; Simmons and Singleton 2008).

Proficient reading is typified by the balance between fast and automatic retrieval of phonological and whole‐word mapping of orthography to semantics (Seidenberg and McClelland 1989). But phonology in particular is critical in the context of reading acquisition, where children must learn to precisely map phonemes (sounds) onto orthographic representations (letters, in alphabetic languages) (Brady and Shankweiler 2013; Compton 2003; Hulme and Snowling 2013; Landerl et al. 2022). Similarly, arithmetic abilities are grounded in the association of written number symbols with their corresponding verbal labels and the precise quantities they represent (Gilmore et al. 2007; Göbel et al. 2014; Östergren and Träff 2013; Wong et al. 2016). Much like reading, the early development of proficient arithmetic abilities is characterized by changes in which procedural strategies involving explicit numerical manipulations are eventually replaced, albeit not completely, with faster, automatic retrieval of arithmetic facts from memory (Geary et al. 1996; Siegler 1987, 1988). Critically, according to the Triple Code Model of mathematical abilities these memorized arithmetic facts are represented in the brain according to their phonological and lexical attributes along the language processing network, just like the representations of written words (Dehaene 1992; Dehaene et al. 2004; Dehaene and Cohen 1995, 1998). As a result, an individual's ability to encode, retrieve, and manipulate phonological representations with precision may influence not only reading development, but also the development of verbal strategies for arithmetic. A growing body of behavioral and neuroimaging research has found some support for this idea, but the findings remain inconclusive.

### 1.1 Behavioral Evidence of a Link Between Phonological Processing Abilities and Arithmetic

Phonological processing—that is, the manipulation of the sound structure of language (Torgesen et al. 1994; Wagner and Torgesen 1987)—is an important precursor of reading acquisition (Brady and Shankweiler 2013; Compton 2003). This general term encompasses three main components: *phonological awareness*—the ability to distinguish and manipulate smaller units of sound; *phonological memory*—the ability to retain and process verbal sequences in short‐term memory; and *rate of access*—the speed at which individuals can retrieve phonological information from long‐term memory (Liberman 1971, 1973; Scarborough and Brady 2002; Torgesen et al. 1994).

Recent studies have shown that these abilities may also represent an important precursor of arithmetic abilities. For example, phonological awareness contributes uniquely and significantly to arithmetic performance in school‐aged children, even after controlling for covariates such as age, intellectual ability, and working memory (Fuchs et al. 2005; Greiner de Magalhães et al. 2021; Vanbinst et al. 2020; Vukovic and Lesaux 2013). Longitudinally, phonological awareness abilities measured in grade two explain nearly 10% of growth in arithmetic abilities by grade 5, after controlling for previous math abilities and other general factors (Hetch et al. 2001). Moreover, phonological awareness explains the shared variance between reading and math abilities. That is, rather than simply correlating with both domains, phonological awareness helps account for *why* reading and math are correlated (Child et al. 2019) and has been identified as a common risk factor for the co‐occurrence of math and reading difficulties (Snowling et al. 2021). Phonological memory, on the other hand, has also been linked to arithmetic performance, but to a lesser degree (Peng et al. 2016; Trbovich and LeFevre 2003; but see Fuchs et al. 2006; Matejko et al. 2022; Slot et al. 2016). Finally, the rate of access to phonological representations, measured using rapid automatized naming (RAN) tasks, has shown a more consistent association with arithmetic fluency in school‐aged children, both concurrently (Cui et al. 2017) and longitudinally (Koponen et al. 2016).

Critically, individual differences in phonological abilities are not broadly associated with all aspects of arithmetic, but are rather specifically linked to the use of verbal retrieval strategies (Boets and De Smedt 2010; Matejko et al. 2022; Vukovic and Lesaux 2013). Unlike procedural strategies, which involve step‐by‐step methods such as counting, decomposition, and other transformations, retrieval strategies in the context of arithmetic refer to the direct recall of answers from memory (Geary et al. 1996; Siegler 1987, 1988). The association between a problem and its answer is established in long‐term memory through repeated practice, which is why retrieval is typical for simpler, smaller problems that are more familiar and for multiplication problems that are explicitly taught to retrieve from memory (Campbell and Xue 2001; Zbrodoff and Logan 2005). Previous studies have found that both children and adults with formal diagnoses of dyslexia use arithmetical retrieval strategies to a lesser degree and less efficiently than typically developing peers (Boets and De Smedt 2010; De Smedt and Boets 2010). Moreover, phonological awareness in particular is a significant predictor of performance during small arithmetic problems (i.e., where the sum of both terms is 25 or less), but not during problems using larger numbers that require procedural strategies (De Smedt et al. 2010; Matejko et al. 2022). This is again consistent with the proposals of the Triple Code Model, which suggest that verbal processing is more important for overlearned, automatized arithmetic problem solving, whereas procedural strategies may rely more on magnitude processing mechanisms and executive functions.

In summary, there is a growing body of evidence suggesting that precursors of word reading abilities such as phonological processing also play a selective role supporting arithmetic abilities, and more specifically the use of retrieval strategies. However, there are also reports in the literature that fail to observe these specific correlations (Amland et al. 2021; Landerl et al. 2009; Passolunghi et al. 2007; Purpura et al. 2011) and that argue that the unique contributions from phonological processing abilities to arithmetic found in previous studies might stem from an insufficient inclusion of domain‐general predictors as control variables (Jöbstl et al. 2023; Ünal et al. 2023). This diversity in findings underscores the need for further investigation to disentangle the nuanced role of phonological processing in the development of arithmetic abilities.

### 1.2 Neuroimaging Studies About the Link Between Phonological Processing and Arithmetic

The behavioral evidence discussed above, albeit diverse, is consistent with the idea that the ability to store, access, and manipulate phonological representations is important for the development of efficient verbal arithmetic strategies. This idea aligns with the predictions of the Triple Code Model, suggesting that arithmetic facts are stored using a verbal code along the canonical language‐processing network (Dehaene and Cohen 1995). A hypothesis that can be derived from this proposal is that the brain regions responsible for the processing of phonological information are also recruited during arithmetic problem solving. Alternatively, arithmetic could be supported by regions near the perisylvian language network that are anatomically close, yet functionally independent of those involved in phonological processing. Distinguishing between these possibilities is important to understand the association between language and arithmetic abilities in typical populations and advancing neurocognitive models of the frequent co‐occurrence of arithmetic and reading difficulties.

Current neuroimaging evidence has lent some support to the possibility that arithmetic and phonological processing converge along the left temporo‐parietal cortex. For example, the supramarginal and angular gyri are strongly implicated in phonological processing during reading (Graves et al. 2023; Price 2012), but also consistently recruited during arithmetic tasks, especially those requiring the retrieval of overlearned facts rather than procedural computation (De Smedt et al. 2011; Grabner et al. 2009; Polspoel et al. 2017). Moreover, a recent meta‐analysis by Pollack and Ashby (2018) summarizing a large number of neuroimaging studies of either arithmetic or phonological decoding tasks suggests that both processes may also converge along a broader network, including the frontal, insular, and fusiform cortex in children; as well as the inferior parietal and inferior frontal cortex in adults. However, without a direct comparison within participants, claims of overlapping activation remain speculative; especially if we consider the functional complexity of the regions where co‐activation has been described—for example, the inferior frontal gyrus (IFG), where highly specialized language areas lie side‐by‐side with general‐domain regions (Fedorenko et al. 2012).

Nearly 20 years after the proposal of the hypothesis of overlapping mechanisms, a strong body of neuroimaging literature directly comparing the network that supports phonological processing of written words and arithmetic *concurrently* has been severely lacking, with the exception of only a handful of studies (Andin et al. 2015; Evans et al. 2016; Fedorenko et al. 2011; Prado et al. 2011; Simon et al. 2002). The findings from this small body of literature are far from conclusive, and integrating them remains challenging due to substantial variability in experimental paradigms, task demands, and analytic approaches. One of the earliest attempts to test cross‐domain overlap was done by Simon et al. (2002), who examined brain activation using functional magnetic resonance imaging (fMRI) during a subtraction and a phoneme awareness task in which participants judged whether visually presented words contained a target phoneme. This study identified distributed networks associated with both arithmetic and phoneme awareness that overlapped along a specific cluster on the left temporo‐parietal cortex. Similarly, a more recent study by Evans et al. (2016) found evidence of converging activation along the left IFG for addition and word reading (measured using an implicit reading task in which participants detected if a tall letter was present in visually presented words) in a sample including a wide range of ages, from adolescence to adulthood.

Another study by Prado et al. (2011) employed a visual word rhyming paradigm, alongside an arithmetic task that included not only subtraction but also multiplication problems, which allowed to test differences in the recruitment of areas associated with phonological processing during two arithmetic conditions that are typically characterized by the use of procedural vs. retrieval strategies, respectively. Only a cluster—the left middle temporal gyrus—along the network of brain areas recruited during the rhyming task displayed greater activation for multiplication compared to subtraction; but beyond these relative differences, they were not able to replicate the overlap observed by Simon et al. (2002) or the more recent study by Evans et al. (2016), since conjunction analyses at the group level did not show any significant regions of overlap.

Further evidence for distinct mechanisms was provided by Andin et al. (2015), who did not directly test the potential overlap between phonology and arithmetic, but rather compared the degree to which different regions of a predefined language network were recruited during subtraction, multiplication, and a task involving the rhyming of lexical labels corresponding to letters. All three experimental conditions generated significant activation along temporo‐parietal and inferior frontal cortex, but the specific subregions involved in each task were regionally differentiated. Therefore, unlike previous findings, this study supports the idea of anatomically proximal but different mechanisms involved in arithmetic and phonological aspects of reading.

In summary, although several studies have explored the neural correlates of arithmetic and phonological processing independently, direct evidence regarding the extent to which these processes rely on overlapping brain regions remains limited. As a result, it remains an open question whether the apparent convergence in temporoparietal and frontal regions reflects overlapping activation or merely spatial proximity between functionally distinct systems.

### 1.3 The Current Study

In the current study, we used functional magnetic resonance imaging (fMRI) to test the hypothesis that the retrieval of arithmetic facts recruits brain regions that are at least partially overlapping with those involved in phonological processing.

To obtain brain activity specifically associated with phonological subprocesses in reading, participants completed a rhyming judgement paradigm with visually presented words. While rhyming paradigms using nonwords emphasize sublexical orthography‐to‐phonology mapping processes, rhyming tasks with known words have also been used to study the processing of phonological information (Brozdowski and Booth 2021; Cao et al. 2008; Grossi et al. 2001; Hoeft et al. 2007; Lytle et al. 2020). Moreover, we carefully controlled the orthographic (dis)similarity between the words presented so that rhyming judgments could only be performed using the sound information from the words. Thus, while this task is expected to engage a broad set of cognitive and neural mechanisms, it emphasizes phonological segmentation processes to decompose words into individual phonemes and phonological working memory to maintain and manipulate these representations during the rhyming judgment.

We tested the overlap of the brain regions involved in this task with those involved in arithmetic problem solving, using an addition paradigm. Behavioral evidence suggests phonological processing abilities are mainly associated with the retrieval of arithmetic facts from memory—as opposed to using other strategies, such as counting, decomposing, and so forth. However, collecting trial‐specific procedural vs. retrieval strategy reports while participants lie in the scanner is a challenging task. In the absence of explicit strategy data, we manipulated problem size to create conditions that would elicit retrieval to different degrees. Namely, our arithmetic task only included single‐digit addition problems, which we classified into small (sum < 10) or large problems (sum > 10), a distinction that has been previously used to study differences in strategy use (Barrouillet et al. 2008; Campbell and Xue 2001; De Smedt et al. 2010; Imbo and Vandierendonck 2008).

Finally, it is possible that phonological processing and arithmetic overlap across different regions in children versus adults. Although most of the behavioral evidence suggesting a link between precursors of word reading—such as phonological processing—and arithmetic comes from studies in school‐aged children, similar relationships have also been reported in adults (De Smedt and Boets 2010). However, since both arithmetic and reading abilities undergo important developmental changes (Martin et al. 2015; Peters and De Smedt 2017; Schlaggar and Church 2009), the convergence of both domains across similar regions of the brain may change over time, as suggested by recent meta‐analytic evidence (Pollack and Ashby 2018); not only due to developmental changes but also differences in skill level.

We applied two analytic approaches. First, we performed a conjunction analysis to identify brain regions jointly recruited across both tasks. We then used representational similarity analysis (RSA; Kriegeskorte 2008; Kriegeskorte and Diedrichsen 2019) to compare the fine‐grained multivariate patterns of neural activity associated with phonological processing and arithmetic within each participant, to determine the similarity among the two. This type of multivariate analysis might provide critical information to interpret conjunction effects (Peelen and Downing 2007), but also, since similarity estimates can be acquired for each participant, represents a measure of overlap within each participant.

In summary, the current study aims to answer the following questions: (1) Do arithmetic and phonological processing recruit overlapping brain regions? (2) If so, is the pattern similarity between phonological processing and arithmetic different for small versus large problems? Findings from previous studies suggested the inferior frontal, temporal, and inferior parietal cortex as the areas where these processes may converge. Moreover, given the previous evidence suggesting that retrieval strategies are more frequent during small problems, we expect to find greater similarity between this condition and phonological processing compared to large problems, which should be more likely to involve a mix of strategies like retrieval, counting, or decomposition. We expect our findings to contribute to a better understanding of the relationships between phonological processing and arithmetic at the neural level, and, therefore, inform neurocognitive accounts of the observed shared developmental trajectories of math and reading development. In the long run, this knowledge may also contribute to designing interventions that can target those co‐occurring difficulties more effectively.

## 2 Methods

### 2.1 Participants

A group of mothers and their children were recruited in the city of London, Ontario in Canada as part of a larger project aimed to investigate the neurocognitive correlates of arithmetic and reading abilities, as well as the intergenerational influences in the development of these skills. A full description of the project goals and design can be found here: https://osf.io/e2mq5/. The adult sample initially included 35 healthy adults and 42 children between 4th and 9th grade (in some cases, multiple children of the same mother were included). One of the adult participants had to be removed from the final analysis due to acquisition issues that prevented the preprocessing of their data. One child participant had to be excluded due to excessive motion during the fMRI acquisition session. The final group included 41 children (17 female, average age = 12.34 years, age range = 10–14 years) and 34 mothers (all female, mean age = 44.12, age range = [34, 55]). All participants were fluent English speakers with normal or corrected vision and no history of neurological disease and no reports of reading or math difficulties.

### 2.2 fMRI Task Description

**FIGURE 1:**
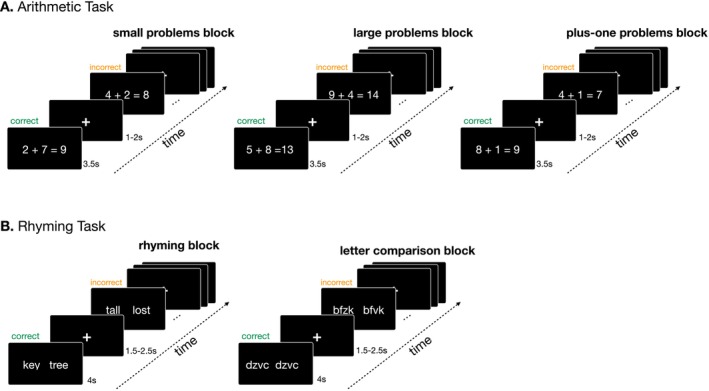
fMRI experimental design and example blocks from each task.

Children and adults completed the same neuroimaging paradigm, which included two runs of an arithmetic task and two runs of a visual rhyming judgment task (Figure 1).

The design of the arithmetic task follows the paradigm developed by Matejko and colleagues, which has been extensively validated to investigate the neural network supporting arithmetic in both children and adults (Matejko and Ansari 2017, 2019, 2021). Participants were presented with single‐digit addition problems and a solution (e.g., 2 + 3 = 5) and were instructed to press a button if the presented solution was correct, and a different button if it was incorrect. Incorrect trials included solutions that were calculated as the correct solution +1 or +2 (e.g., 3 + 9 = 13 or 5 + 7 = 14); −1 or −2 solutions were avoided to ensure that the incorrect answer was never equal to or smaller than one of the addends (e.g., 7 + 2 = 7 or 5 + 1 = 4) (Matejko and Ansari 2019). The problems presented were organized in three conditions: small problems (sum < 10), large problems (sum > 10). As a control for low‐level visual features, we use a plus‐one condition that also included single‐digit addition problems, but one of the addends was always 1. This type of plus‐one problems is typically solved using simple counting procedures, also known as “the number after” rule (Baroody 1995; LeFevre et al. 1996), which are different from procedural or retrieval strategies. Therefore, this condition represents an effective control that matches the low‐level features of the experimental conditions almost perfectly, with minimal cognitive demands. Each arithmetic run included two blocks of 6 trials for each experimental condition. On each trial, stimuli remained on the screen for 3.5 s; inter‐trial intervals were varied at random and could have a duration of 1, 1.5, or 2 s. The order of conditions and the trials included in each block were randomized across participants. Arithmetic blocks had a duration of 30 s; inter‐block fixation intervals lasted on average 9 s (jitter interval of 8.6–9.4 s), and the total duration of this task was 244 s.

The rhyming judgment task included two conditions: rhyming and letter matching. During the rhyming condition, participants judged whether two visually presented words rhymed (e.g., *light*—*bite*) or not (e.g., *deep*—*help*) by pressing two different buttons. The two words were presented simultaneously on the screen. The words included in each pair always had conflicting orthography, regardless of whether they rhymed. This was done so that participants could not use visual information to make rhyming judgments. The letter matching condition was used to control for visual and motor affordances; in this task participants determined whether two unpronounceable consonant strings (e.g., *pcvh*—*psvh*) visually matched. There were three blocks of the rhyming condition (5 trials per block) and three blocks of a letter‐matching control condition (5 trials per block). On each trial of either condition, stimuli remained on the screen for 4 s; inter‐trial intervals were varied and could have a duration of 1.5, 2 or 2.5 s. The presentation order of the blocks was randomized across participants, as well as the order of trials included on each block. Each block had a duration of 30 s; inter‐block fixation intervals were averaged to 9 s in each run (jitter interval of 8.6–9.4 s), and the task had a total duration of 244 s.

### 2.3 fMRI Acquisition and Preprocessing

All the imaging data was collected on a 3 T Siemens Prisma Fit scanner using a 32‐channel head coil. The sequences were identical for the reading and arithmetic tasks: TR = 1 s, number of slices = 48, number of volumes = 244, voxel size = 2.5 × 2.5 × 2.5 mm, multiband acceleration factor = 3, flip angle = 40°, and iPAT accelerator factor PE = 2. The effect of head motion on data will be determined by preprocessing all fMRI data using the Artifact Detection Tools (ART) toolbox (Whitfield‐Gabrieli and Nieto‐Castanon 2012), which analyzes both frame‐wise displacement (i.e., jump motion) and volume‐to‐volume deviation of the global BOLD signal across the brain. Excessive frame‐wise displacement was defined as exceeding 1.5 mm, and excessive deviation was defined as a mean volume intensity four standard deviations beyond the z‐normalized global signal across runs. Volumes that exceed these thresholds in either category (i.e., displacement or signal) were flagged as outliers. If 20% of an individual's volumes in a run were marked as outliers, that run was removed from further analysis.

The functional data that were included in the analysis was preprocessed using fmriprep (Esteban et al. 2018) and SPM (Friston et al. 2007). Images were corrected for head motion, linear trends, and low‐frequency noise. Functional images were coregistered to the T1‐weighted anatomical images and normalized to the standard Montreal Neurological 152‐brain average template. For all univariate analyses, data was spatially smoothed using a 6 mm FWHM Gaussian smoothing kernel. Motion realignment parameters were included as regressors of no interest in the General Linear Model to further control for variation due to movement artifacts. An additional covariate of no interest was created to control for outlying volumes identified by ART (i.e., volume‐to‐volume motion > 1.5 mm or > 4SD mean signal deviation).

### 2.4 fMRI Analysis

#### 2.4.1 Conjunction Analysis

Our analysis plan was preregistered in the Open Science Framework and can be found at https://osf.io/rt875. Univariate analyses were conducted using the Statistical Parametric Mapping toolbox in Matlab (Friston et al. 2007). We used a General Lineal Model at the individual level that included four runs, two corresponding to the arithmetic task and two corresponding to the rhyming task. On each run, we included separate regressors for each condition (Small, Large, Plus One for the arithmetic task; Rhyming, Letter Matching for the rhyming task) and one regressor to model incorrect trials (if any). Nuisance covariates included six head motion parameters modeling translation and rotation across the *X*, *Y*, and *Z* as well as a regressor of high‐movement volumes identified with the ART Toolbox (Whitfield‐Gabrieli and Nieto‐Castanon 2012).

Brain activation corresponding to arithmetic was obtained with the contrast: [Small + Large > Plus One] (i.e., the average activation obtained during the Small and Large problems was contrasted against the Plus‐one control). Brain activation associated with phonological processing was obtained with the contrast: [Rhyming > Letter Matching]. Regions of overlapping activation for reading and arithmetic (voxels in which both contrasts were significant) were identified with a second‐level conjunction analysis on each group separately. The conjunction maps were initially defined using an uncorrected *p* < 0.001 for both contrasts and then cluster‐corrected. The parameters for the cluster correction were estimated separately for children and adults using the 3dClustSim algorithm on AFNI (with a cluster forming threshold of 0.05) (Cox et al. 2017). That cluster threshold was 35 voxels for the children group and 18 voxels for the adult group.

#### 2.4.2 Representational Similarity Analysis

A multivariate pattern analysis was performed to determine if there were differences in the similarities between the rhyming condition and each of the arithmetic problem types. For each participant, we defined regions of interest (ROI) based on the clusters where significant conjunction effects were observed. For each ROI, we extracted the distributed patterns of activity corresponding to each condition of interest (Small, Large, Rhyming) contrasted against fixation. These patterns were then vectorized, and pair‐wise similarities between conditions (Small‐Large, Small‐Rhyme, Large‐Rhyme) were estimated using Pearson's Correlation Coefficients, which were then normalized using Fisher's transformation.

In designing the statistical test to contrast the Small‐Rhyme to the Large‐Rhyme similarity value, we had to account for the fact that these two coefficients are not independent. Moreover, if the neural patterns for the Small and the Large conditions were very similar to each other in the first place, that would hinder the ability of any test to find any differences in the way each of them correlates with a third condition. With that in mind, we selected Steiger's *Z* (Steiger 1980) as a more robust statistic to assess the difference between the two similarity values on each ROI. This test was designed specifically to compare two correlation values coming from the same sample that also have a variable in common and account for its inter‐dependency. At the group level, we tested whether the average *Z* coefficient for a given ROI was significantly different from 0 using a *t*‐test. The significance of these tests was assessed at an uncorrected alpha = 0.05 and then corrected for multiple comparisons using a Dunn–Šidák correction (Šidák 1967).

We also examined if each similarity value was significantly different from 0. It is known that correlations between high‐dimensional neural activation patterns are typically influenced by elements such as shared noise or global signal components that often lead to a nonzero mean correlation under the null hypothesis (Cai et al. 2019; Diedrichsen et al. 2021; Kriegeskorte et al. 2006; Kriegeskorte and Douglas 2019; Ritchie et al. 2017, 2021; Walther et al. 2016). Procedures such as multivariate noise and cross‐validation further prevent overfitting and provide similarity estimates with a meaningful zero baseline, making them suitable for statistical inference (Haxby et al. 2001; Nili et al. 2020; Ritchie et al. 2017; Walther et al. 2016). Therefore, for this part of the analysis, we re‐estimated the pairwise similarity between our conditions of interest including these procedures to make sure the observed significant correlations were not inflated by noise. A detailed description of this part of the analysis is included in the S1 A.

## 3 Results

### 3.1 fMRI Task Performance

**FIGURE 2:**
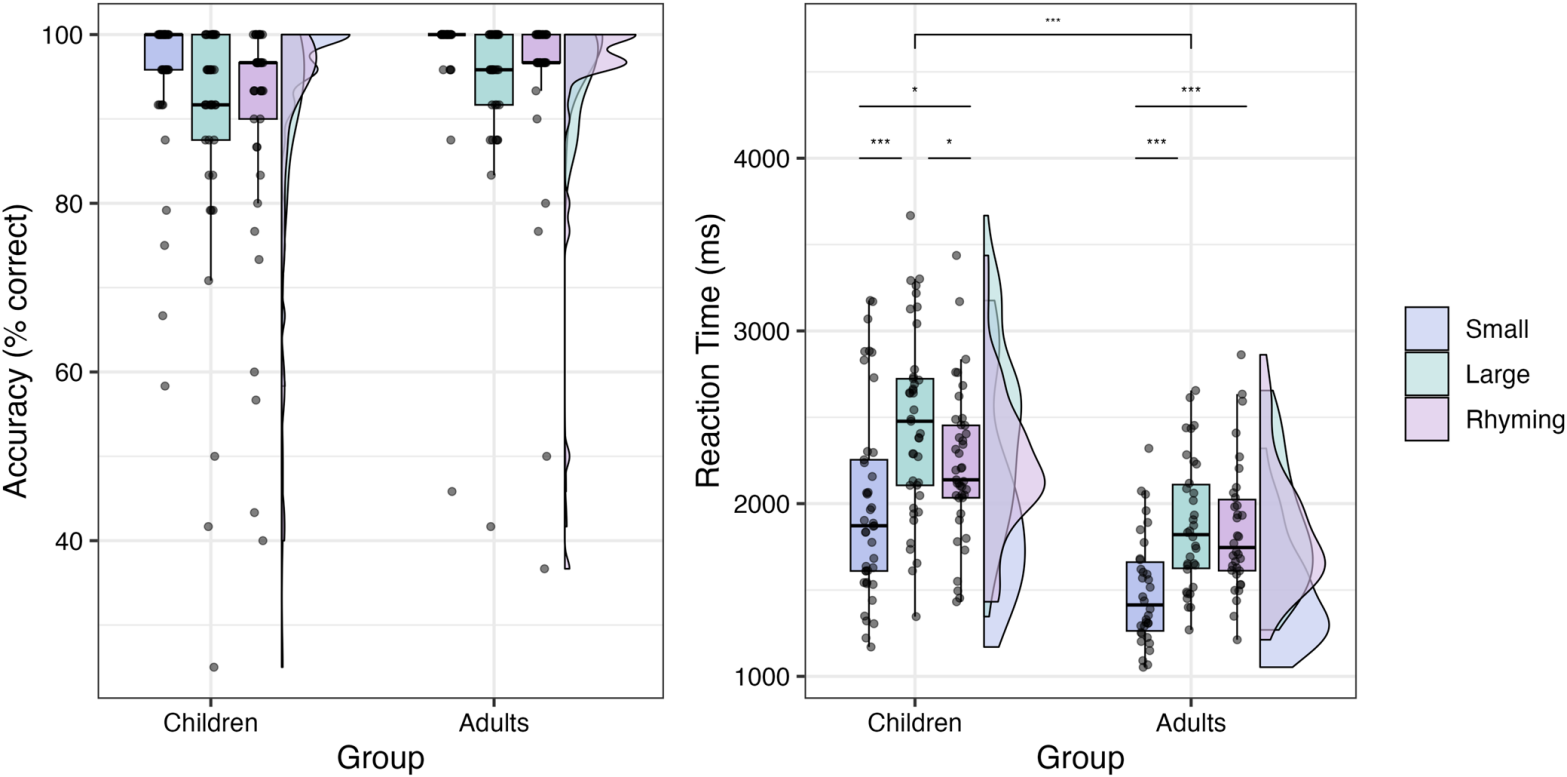
Distribution of accuracy and reaction time data for fMRI tasks. The horizontal black line in the middle of each boxplot represents the group median; the lower and upper hinges correspond to the first and third quartiles; the upper and lower whiskers extend from the hinges to the largest value no further than ±1.5 × Inter Quartile Range. To the right of the boxplots, we have represented a smooth density distribution of the data. Asterisks represent significance of the differences between means; '*' : *p* < 0.05, '**' : *p* < 0.01; '***' : *p* < 0.001.

Both children and adults displayed high levels of task accuracy for all conditions, especially for small problems (Figure 2). In terms of reaction times, we found significant effects of both group (*F*(1, 73) = 28.30, *p* < 0.001, *η*2 = 0.03) and condition (*F*(1.42, 103.44) = 51.43, *p* < 0.001, *η*2 = 0.138). On average, adults responded faster than children (*t* = 7.76, *p* < 0.001). In addition, children were faster at the small problems compared to both the large problems (*t* = −11.44, *p*_adj_ < 0.001) and the rhyming task (*t* = −3.01, *p*_adj_ = 0.013); and faster at the rhyming task compared to the large problems (*t* = 2.95, *p*_adj_ = 0.016). Adults were faster responding to small problems compared to both the large problems (*t* = −12.15, *p*_adj_ < 0.001) and the rhyming task (*t* = 7.93, *p*_adj_ < 0.001).

### 3.2 fMRI Data

#### 3.2.1 Analysis 1: Overlap Between Arithmetic and Rhyming

##### 3.2.1.1 Adults

Brain activation related to arithmetic was modeled using both the small and large problems, contrasted against the plus‐one condition as a control (Small + Large > Plus One). For the adult group, this contrast revealed bilateral clusters along frontal, parietal, and occipitotemporal cortex. The activation related to phonological processing was estimated using the rhyming condition contrasted against the letter matching as a control (Rhyming > Letter Matching). This task recruited a network of mostly left‐lateralized regions, including mainly frontal and temporo‐parietal cortex. A detailed description of the areas of significant brain activation observed for each task separately can be found in the S2 B.

**FIGURE 3:**
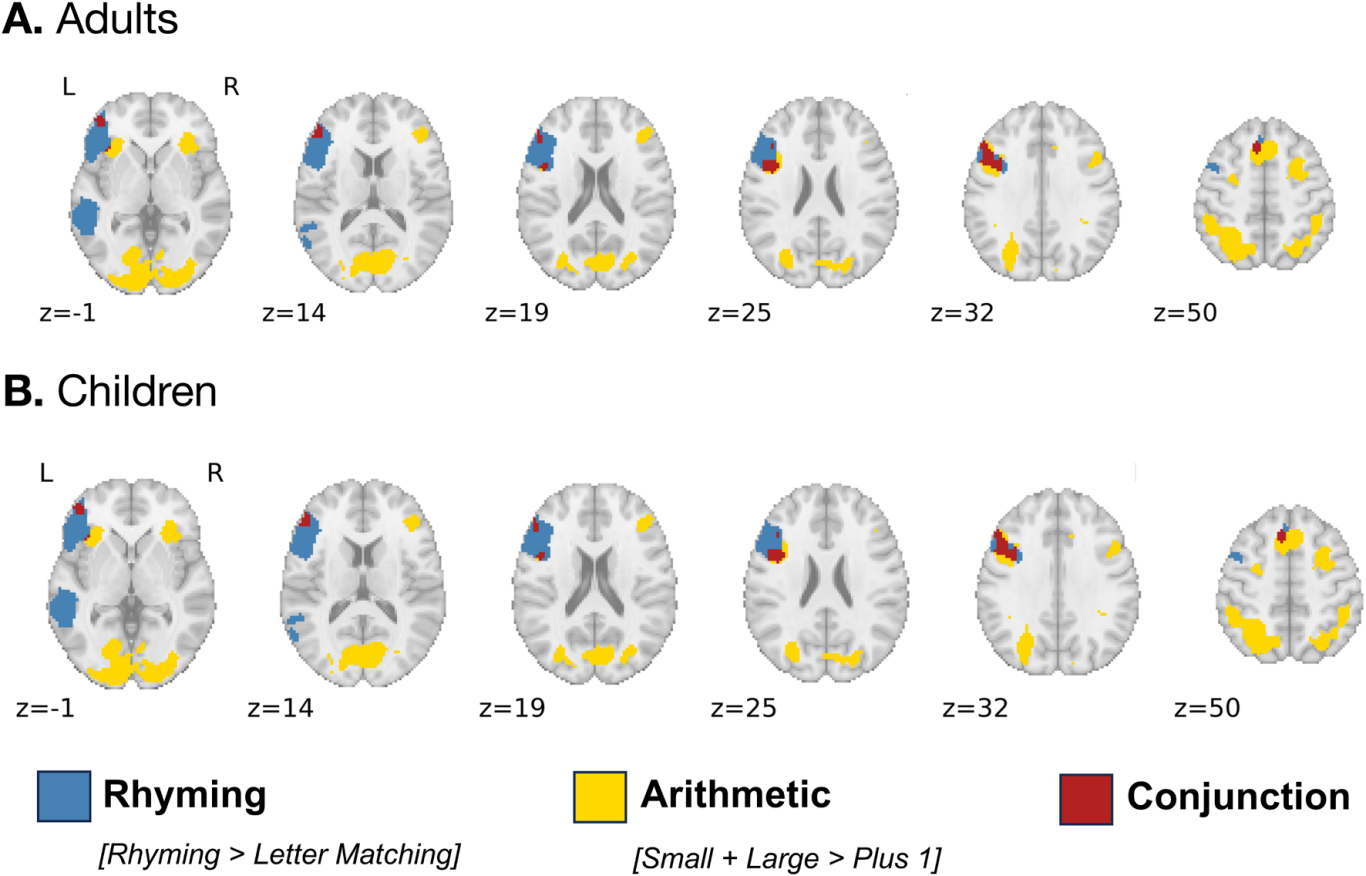
Conjunction analysis of the brain activation for the Arithmetic and Rhyming tasks. *t*‐maps for each task were thresholded at *p* < 0.001 and conjunction results were cluster corrected at 18 and 35 voxels for adults and children, respectively.

The conjunction between these separate contrasts revealed three clusters of significant overlap between the rhyming and the arithmetic task (Figure 3A; Table 1): one along the left inferior and middle frontal gyrus (IFG), a second cluster on the right posterior cerebellum (CBL), and a third cluster on the left inferior temporal gyrus (ITG).

**TABLE 1:** Clusters resulting from the conjunction between Arithmetic and Rhyming.

Group	Cluster	*k*	Peak coordinate	Description
Adults	IFG	264	[−48, 36, 14]	254 voxels in left Inferior Frontal Gyrus—pars triangularis 6 voxels in left Middle Frontal Gyrus 3 voxels in left Inferior Frontal Gyrus—pars opercularis 1 voxel in left Inferior Frontal Gyrus—pars orbitalis
	CRB	21	[10, −80, −28]	21 voxels in right Posterior Cerebellum (Crus I, Crus II)
	ITG	31	[−58, −47, −13]	22 voxels in left Inferior Temporal Gyrus 9 voxels in left Middle Temporal Gyrus
Children	IFG1	235	[−49, 12, 29]	89 voxels in left Inferior Frontal Gyrus—pars opercularis 66 voxels in left Precentral Gyrus 59 voxels in left Inferior Frontal Gyrus—pars triangularis 21 voxels in left Middle Frontal Gyrus
	IFG2	44	[−51, 39, 17]	40 voxels in left Inferior Frontal Gyrus—pars triangularis 2 voxels in left Middle Frontal Gyrus
	SFG	46	[−7, 24, 49]	27 voxels in left Supplementary Motor Area 19 voxels in the left Medial Frontal Gyrus
	MFG	49	[−46, 49, 2]	31 voxels in left Middle Frontal Gyrus 12 voxels in left Inferior Frontal Gyrus—pars triangularis 6 voxels in left Inferior Frontal Gyrus—pars orbitalis

##### 3.2.1.2 Children

Brain activation associated with arithmetic and phonological processing was estimated in the children group using similar contrasts as the ones described above. For the arithmetic task, we found significant activation across bilateral clusters along the frontal, parietal, and occipital cortex. The areas recruited for phonological processing were almost exclusively left‐lateralized. A detailed description of the areas of significant brain activation observed for each task separately can be found in the S3 C.

The conjunction of these two univariate activation maps revealed 4 clusters of significant overlap in children (Figure 3B; Table 1): two of them on the inferior frontal gyrus (IFG1, IFG2), one cluster on the middle frontal gyrus (MFG), and a last one on the medial surface of the superior frontal gyrus (SFG).

#### 3.2.2 Analysis 2: Is Problem Size Important?

To examine the role of arithmetic problem size on the observed overlap, we first examined if there were differences in brain activation for each problem type separately. In addition, we tested the correlations between the neural patterns associated with each problem type and neural patterns associated with phonological processing.

**FIGURE 4:**
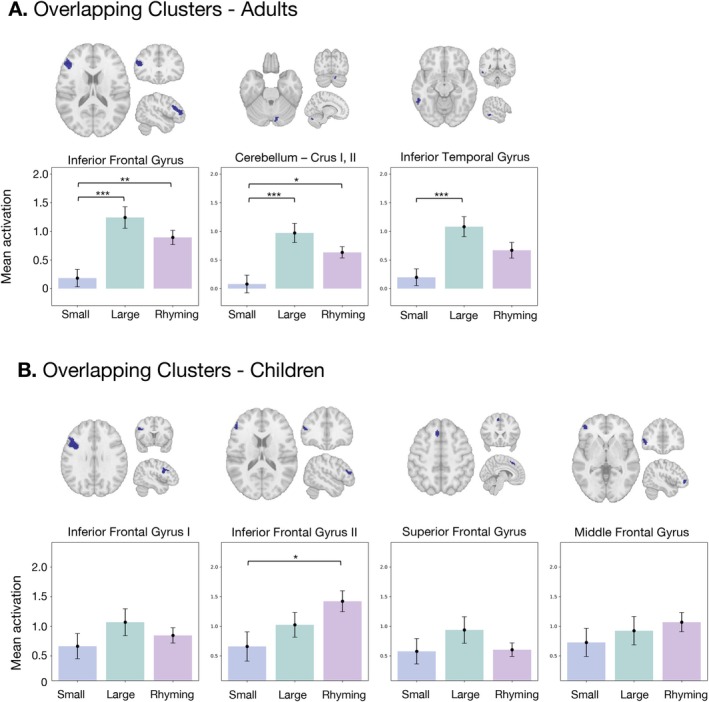
Differences in average activation between conditions on the clusters of significant overlap between arithmetic and rhyming. *p < 0.05, **p < 0.01, ***p < 0.001. CRB: right posterior cerebellum; IFG: inferior frontal gyrus; ITG: inferior temporal gyrus.

To test if there were differences in brain activation between our experimental conditions (Small, Large, Rhyming), we ran a repeated measures ANOVA model on each cluster. In the adults (Figure 4A), we found a significant effect of condition in all the clusters (IFG: *F*(2, 66) = 13.58, *p* < 0.001, *η*2 = 0.19; CRB: *F*(2, 66) = 13.63, *p* < 0.001, *η*2 = 0.16; ITG: *F*(2, 66) = 11.57, *p* < 0.001, *η*2 = 0.14). Post hoc contrasts revealed that the activation observed during Small problems was significantly lower than the activation observed during Large problems in all the clusters (IFG: Mean_diff_ = −1.06, 95% CI [−1.58, −0.53], *p*_adj_ < 0.001; CRB: Mean_diff_ = −0.89, 95% CI [−1.37, −0.41], *p*_adj_ < 0.001; ITG: Mean_diff_ = −0.88, 95% CI [−1.40, −0.37], *p*_adj_ < 0.001). In addition, the average activation associated with Small problems was lower than the activation associated with Rhyming in the clusters located in the Inferior Frontal Gyrus (IFG: Mean_diff_ = −0.71, 95% CI [−1.24, −0.18], *p*_adj_ = 0.005) and the cluster in the right posterior lobe of the cerebellum (CRB: Mean_diff_ = −0.55, 95% CI [−1.03, −0.07], *p*_adj_ = 0.02).

Similarly, in children (Figure 4B), we found evidence of significant differences between conditions only in the IFG2 cluster (*F*(2, 80) = 4.03, *p* < 0.021, *η*2 = 0.05), where the activation associated with Small problems was lower than the activation related to Rhyming (Mean_diff_ = −0.76, 95% CI [−1.48, −0.05], *p*_adj_ = 0.033).

We also extracted the multi‐voxel patterns of activation for each condition along these clusters. Neural similarity between the Rhyming and each of the arithmetic conditions was computed using Pearson's correlations, and the obtained coefficients were then normalized via Fisher's transformation. To estimate the difference between the neural similarity for rhyming and small problems versus rhyming and large problems on each individual participant, we used Steiger's *Z*, a robust statistical test designed for contrasting dependent correlation coefficients. At the group level, the statistical significance of the obtained Steiger coefficients was assessed using a *t*‐test. In this context, a value of *t* greater than zero suggests greater neural similarity between rhyming and small problems compared to rhyming and large problems. A significant but negative coefficient suggests the opposite pattern (Table 2).

**TABLE 2:** Contrast between the Rhyming~Small and the Rhyming~Large similarities.

Adults					Children				
ROI	Mean Steiger's coefficient	SD	*t*	*p*	ROI	Mean Steiger's coefficient	SD	*t*	*p*
IFG	−4.18	4.39	−**5.48**	< 0.001	IFG1	−1.93	4.28	−**2.85**	0.007
CRB	−0.71	1.20	−**3.41**	0.002	IFG2	−1.25	3.34	−2.37	0.023
ITG	−0.95	1.69	−**3.22**	0.003	SFG	−0.99	2.40	−**2.62**	0.012
					MFG	−0.66	2.57	−1.61	0.115

*Note:* Significant statistic values are shown in bold. Significance was tested using a corrected alpha = 0.017 for Adults and alpha = 0.013 for Children, estimated using Dunn–Šidák correction.

Abbreviations: CRB: right posterior cerebellum; IFG: inferior frontal gyrus; ITG: inferior temporal gyrus; MFG: middle frontal gyrus; SFG: superior frontal gyrus.

**FIGURE 5:**
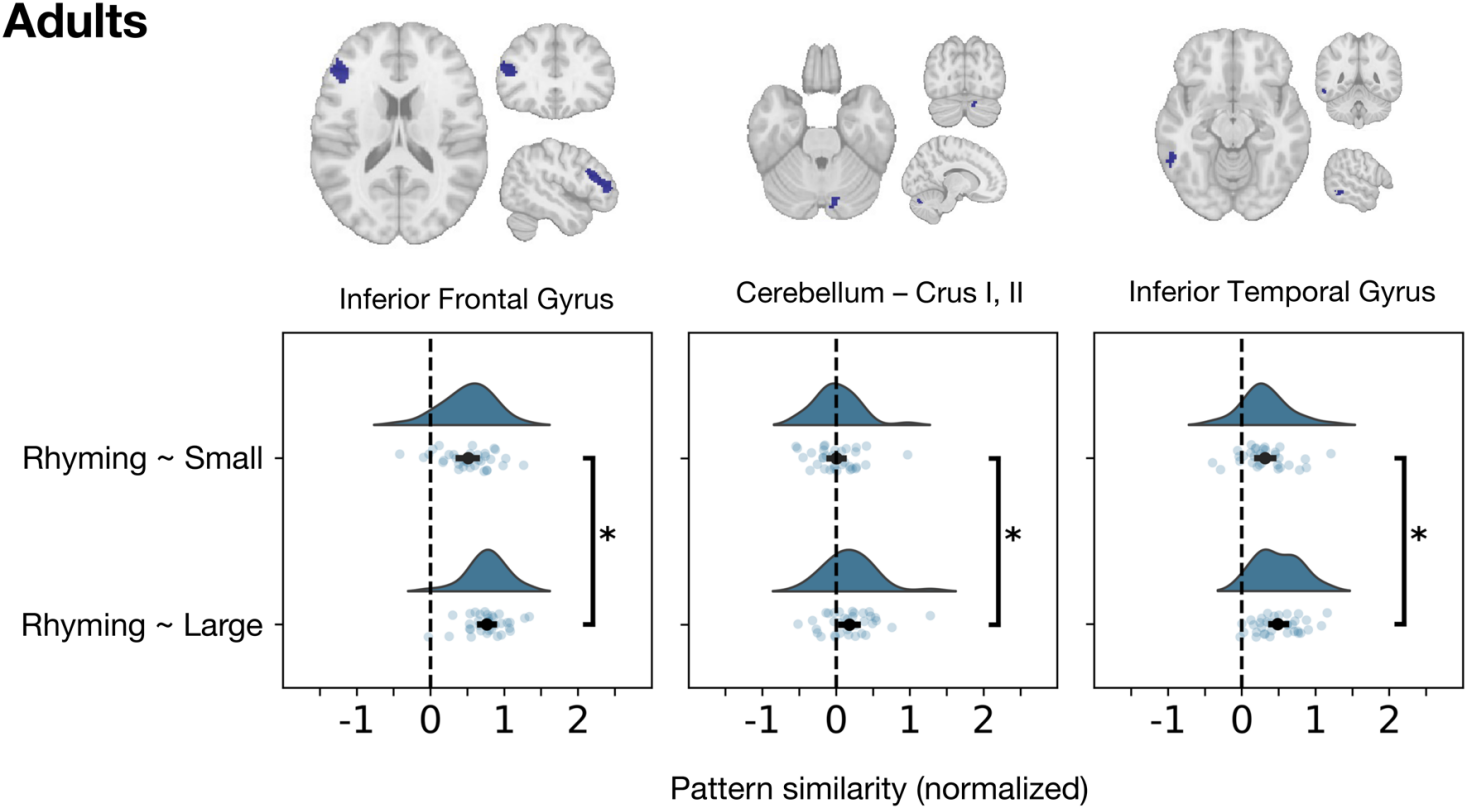
Neural similarity between rhyming and each of the arithmetic conditions, obtained for each overlapping cluster in the Adults. The half‐violin plot on top illustrates the density distribution of the data. Since correlation values have been normalized using Fisher's transformation, their values can extend beyond 1 and −1. The dot below the density plot represents the mean of the distribution; the horizontal lines represent the 95% confidence interval around the mean. The scatterplot shows the individual data points. Significant differences between the means of the similarities for Rhyming~Small and Rhyming~Large are shown using a vertical line next to a * symbol.

The results for the Adults are summarized in Table 2 and Figure 5. Each plot displays the density distribution of the data, the measures of central tendency and variability, and each individual data point. We observed significant differences between the obtained similarity values (Table 2); however, the directionality of the effect was the opposite of our predictions: the neural similarity between the patterns for rhyming and large problems was consistently greater than the similarity between rhyming and small problems. These differences remained significant after correcting for multiple comparisons. For the children, only the cluster in the Superior Frontal Gyrus and one of the clusters in the Inferior Frontal Gyrus displayed significant differences between the similarity values for rhyming and each of the arithmetic conditions (Table 2). Just like in adults, these differences were in the opposite direction to our prediction, favoring neural similarity between phonological processing and large problems and not small problems.

**FIGURE 6:**
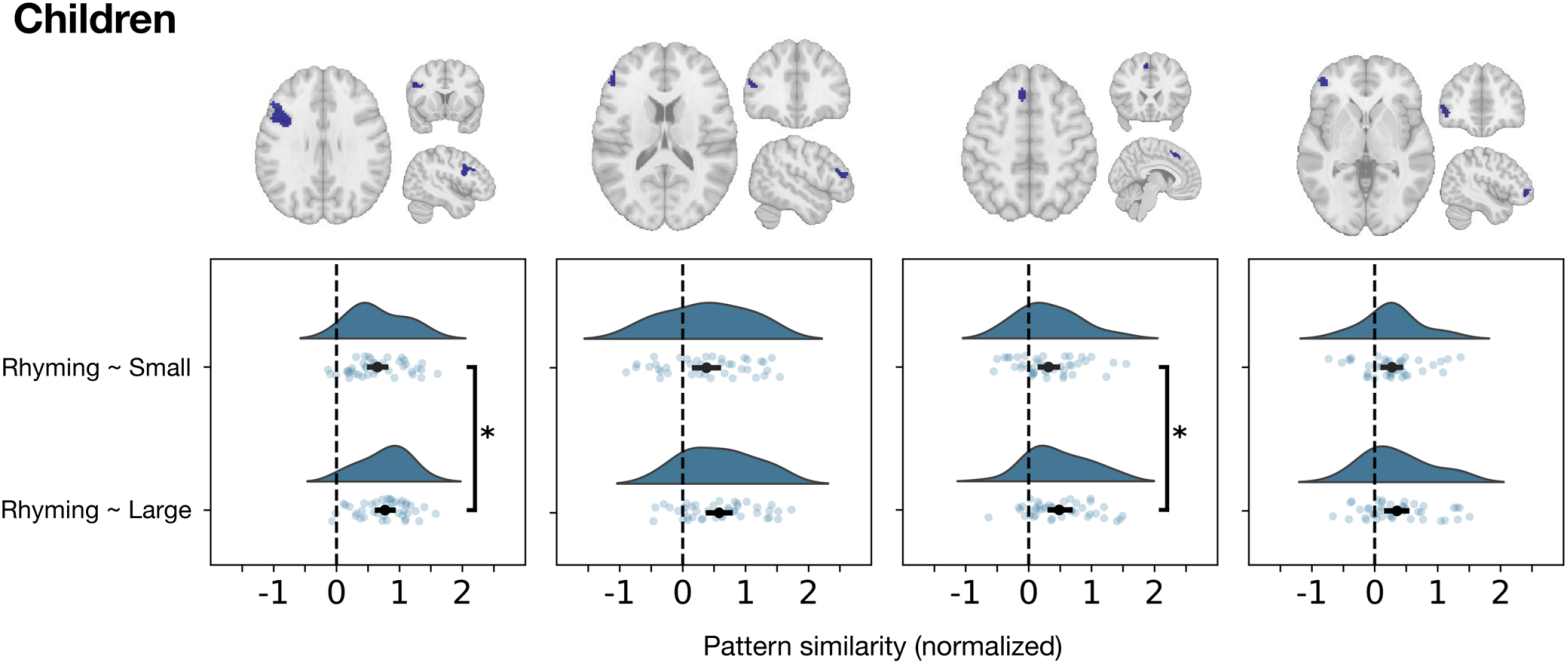
Neural similarity between rhyming and each of the arithmetic conditions, obtained for each overlapping cluster in Children. The half‐violin plot on top illustrates the density distribution of the data. Since correlation values have been normalized using Fisher's transformation, their values can extend beyond 1 and −1. The dot below the density plot represents the mean of the distribution; the horizontal lines represent the 95% confidence interval around the mean. The scatterplot shows all the individual data points. Significant differences between the means of the similarities for Rhyming~Small and Rhyming~Large are shown using a vertical line next to a * symbol.

While this approach is useful to estimate relative differences between two similarity values, it cannot inform whether the similarity values being compared are significant in the first place. That is, this analysis alone cannot tell us if the observed relative differences are, for example, due to the rhyming condition being similar to one of the arithmetic conditions, but not the other; or whether the rhyming condition was similar at the multivariate level to both arithmetic conditions regardless of whether one similarity value is much higher than the other. A visual inspection of the plots shown in Figures 5 and 6 may suggest that, on average, the similarity values obtained may be significantly different from zero. To test if this was the case, we re‐estimated correlation values for each ROI using multivariate noise normalization and split‐half procedures, to avoid obtaining inflated correlation values (S1 A) and conducted a *t*‐test to determine if, on average, each similarity value was significantly different from 0 (Table 3). In the adults, all the similarities observed (Rhyming‐Small, Rhyming‐Large) were significant and positive at the group level, with the exception of the similarity for Rhyming~Small in the right posterior cerebellum. In children, all the pair‐wise similarity values were also, on average, positive and significant (Table 3).

**TABLE 3:** Similarity analysis for the clusters with significant overlap for Arithmetic and Reading using cross‐validated measures.

Group	ROI	Condition	Mean	SD	95% CI	*t*‐test (parametric)		
						*t*	*p*	Cohen's *d*
Adults	IFG	Small‐Rhyming	0.15	0.07	[0.13, 0.18]	**12.47**	< 0.001	−1.10
		Large‐Rhyming	0.18	0.09	[0.15, 0.21]	**11.85**	< 0.001	−1.10
	CRB	Small‐Rhyming	0.01	0.16	[−0.04, 0.07]	0.40	0.6914	−0.01
		Large‐Rhyming	0.14	0.18	[0.08, 0.20]	**4.54**	< 0.001	−0.70
	ITG	Small‐Rhyming	0.16	0.17	[0.10, 0.22]	**5.43**	< 0.001	−0.79
		Large‐Rhyming	0.25	0.16	[0.20, 0.31]	**8.95**	< 0.001	−1.06
Children	IFG1	Small‐Rhyming	0.16	0.08	[0.13, 0.18]	**12.10**	< 0.001	−1.12
		Large‐Rhyming	0.18	0.08	[0.16, 0.21]	**14.63**	< 0.001	−1.12
	IFG2	Small‐Rhyming	0.15	0.18	[0.10, 0.21]	**5.53**	< 0.001	−0.73
		Large‐Rhyming	0.24	0.18	[0.19, 0.30]	**8.49**	< 0.001	−0.95
	SFG	Small‐Rhyming	0.12	0.16	[0.07, 0.17]	**4.84**	< 0.001	−0.63
		Large‐Rhyming	0.17	0.16	[0.12, 0.22]	**6.88**	< 0.001	−0.93
	MFG	Small‐Rhyming	0.10	0.11	[0.07, 0.13]	**5.63**	< 0.001	−0.73
		Large‐Rhyming	0.12	0.15	[0.08, 0.17]	**5.33**	< 0.001	−0.74

*Note:* Significant statistic values are shown in bold. Significance tested using a corrected alpha = 0.002 for Adults and Children estimated using Dunn–Šidák correction.

Abbreviations: CRB: right posterior cerebellum; IFG: inferior frontal gyrus; ITG: inferior temporal gyrus; MFG: middle frontal gyrus; SFG: superior frontal gyrus.

## 4 Discussion

The current study was aimed at testing the hypothesis that arithmetic fact retrieval and phonological processing rely on functionally overlapping brain areas. Previous literature examining each of these processes separately has suggested that both processes involve similar areas of the canonical language network, especially as it relates to phonological processing. But without a consistent body of literature studying both processes concurrently, the possibility that these findings are the result of two networks that are anatomically close, yet functionally independent versus overlapping has remained an open question. We addressed this question using both a test of univariate overlap and a rigorous pattern similarity analysis to provide a more nuanced assessment of brain‐level associations across both domains. Finally, by examining the effect of problem size, we introduced an additional layer of analysis to explore how factors like strategy use may modulate the degree of similarity between the neural patterns associated with arithmetic and phonological processing of visually presented words.

Our findings suggest that: (1) There is a significant overlap between the areas involved in the solution of arithmetic problems and the access to phonological representations of written words as measured with a rhyming task. In the adults, both processes converged along the left inferior frontal gyrus, the left inferior temporal gyrus and the right cerebellum; in the children, we observed overlap along the frontal cortex, including clusters in the superior, middle and inferior frontal gyrus. (2) Within the regions of the brain that displayed significant overlap between arithmetic and rhyme processing, there were also significant linear associations between their distributed patterns of activation, suggesting similarity in the mechanisms recruited across domains. (3) Finally, we did not observe evidence of higher correlations between the patterns evoked in these regions during the word rhyming task and small arithmetic, compared to large arithmetic problems. These findings were in contrast to our predictions that the mechanisms supporting word reading would be more closely associated with the arithmetic condition more likely to involve verbal retrieval (i.e., small problems).

The conjunction analysis presented here represents an important contribution to the literature examining the potential links between arithmetic and word reading by providing direct statistical evidence of the long‐hypothesized overlap between these processes at the neural level. This represents an important contribution to this area of inquiry, where most of the evidence suggesting overlap has come from observations of the neural network supporting arithmetic or phonological processing independently (Pollack and Ashby 2018), with only a few attempts to provide direct tests of overlap (Andin et al. 2015; Evans et al. 2016; Prado et al. 2011; Simon et al. 2002). Unlike Simon et al. (2002), we did not observe coactivation of the inferior parietal cortex for arithmetic and tasks involving access to phonological representations of visually presented words. Only one study we are aware of (Prado et al. 2011) provides results of both conjunction analyses as well as pattern correlations. Unlike our study, however, Prado et al. could not find any significant overlap at the univariate level, but also observed no correlations between the patterns associated with retrieval arithmetic (measured as multiplication > subtraction) and phonological processing. The divergence between these findings and our own suggests the need for further exploration, and it is particularly noteworthy, considering that we used more conservative analytical methods. In particular, the multivariate correlations between the patterns for each arithmetic condition and the rhyming task were estimated using split‐half procedures to ensure that the significant similarity between the patterns was not inflated by correlated noise. This may decrease the reliability of the patterns obtained since only half of the data is used to estimate the patterns for each condition but provides the advantage of more meaningful pairwise correlation values.

### 4.1 Mechanisms Explaining the Convergence Between Arithmetic and Phonological Processing at the Neural Level

While our results suggest that the networks for word reading and arithmetic at least partially overlap, our study alone cannot explain in detail the exact cognitive processes that are responsible for these findings. Previous literature had suggested two main theoretical accounts to explain the possible convergence of both processes along similar regions of the brain (see De Smedt 2018 for a discussion). The most popular account posits that arithmetic fact retrieval, as well as word reading, both rely on phonological representations and that the storage, retrieval, and manipulation of those representations involve similar mechanisms. This theory has also been supported by behavioral evidence showing that phonological awareness uniquely and significantly predicts arithmetic fact retrieval (above other phonological abilities like phonological memory or rapid access). For that reason, most of the small literature exploring relationships between word reading and arithmetic at the neural level has focused on phonological tasks (Andin et al. 2015; Prado et al. 2011; Simon et al. 2002), and motivated our own use of a visual rhyming judgment task. However, considering that our paradigms involved rhyming judgments of words (as opposed to nonwords), and that all our participants were high‐performing, fluent readers, the brain activation observed during this task is probably influenced not only by phonological processing, but also sight word reading mechanisms (with potentially automatic access to semantic representations). Regardless, our central question regarding functional overlap with arithmetic remains relevant. In fact, another account for the potential overlap between word reading and arithmetic poses that both processes are subserved by general symbolreferent mapping mechanisms, through which visual symbols—such as words or arithmetic problems—are associated with their semantic representations (Price and Ansari 2011). This idea is consistent with cross‐linguistic studies of reading showing that the core reading network—encompassing the left inferior frontal gyrus, temporoparietal regions, and occipitotemporal cortex—is consistently recruited across orthographies, including both alphabetic languages that emphasize grapheme–phoneme conversion as well as logographic languages that rely more heavily on whole‐word recognition (Bolger et al. 2005; Rueckl et al. 2015). Thus, the reading network as a whole may support a more general mechanism for mapping visually presented symbols onto their corresponding phonological and semantic representations, and those mechanisms may be responsible both for the automatic retrieval of familiar words, as well as for the fast retrieval of familiar, single‐digit addition problems.

Further studies are needed to discriminate between the phonological access and the symbol–referent mapping accounts. Moreover, our results suggest that arithmetic and word reading may share not only one but multiple regions along the language network. For example, like previous studies (Andin et al. 2015; Evans et al. 2016; Prado et al. 2011), our results suggest that the left inferior frontal gyrus is one of the hubs where these processes converge. Within this region, the subdivisions where overlap was observed differed between children and adults (S4 D). Namely, overlap in the adults was observed along the anterior part of the pars triangularis subdivision; whereas overlap in the children was located instead in the posterior side of pars triangularis, as well as pars opercularis. While our analysis does not include a direct contrast of these conjunction effects between the groups, we generate, based on previous literature, hypotheses about the reasons behind this distinction that future studies could address. Namely, these subdivisions of the inferior frontal gyrus subserve different roles during word reading, with pars opercularis mainly involved in phonological and articulatory aspects of word reading whereas pars triangularis seems more involved in lexical retrieval and semantic processing (Heim et al. 2009; Katzev et al. 2013). It is also known that specific subsections of both of these subdivisions are differently influenced by task demands across a wide range of cognitive domains, including reading, math, and working memory (Fedorenko et al. 2011; Katzev et al. 2013). It is possible that arithmetic and word reading share similar mechanisms of lexical access, phonological processing or overall task demands and more importantly, that their role changes across development. Future studies could address these questions by including word reading paradigms that allow a better isolation of each of these components and testing their role during arithmetic across a wide range of ages.

We also observed convergence of arithmetic and reading in adults along the ventral temporal cortex, specifically on the middle and inferior temporal gyrus. The location of the cluster identified here matches approximately a region in the ventral occipitotemporal cortex that is known for its preferential response to numbers and arithmetic facts (Grotheer et al. 2018; Yeo et al. 2017). Due to its preferential response to numerals, a cluster near this area is often described as the Number form Area (Merkley et al. 2019; Shum et al. 2013; Yeo et al. 2017). But more generally, the role of the inferior temporal gyrus in arithmetic seems to go beyond just the identification of numerals. For example, the response of this region increases as participants read arithmetic equations, with the higher response to the numerals representing the answer of the problem regardless of whether the equation is presented using numerals or number words (Hermes et al. 2017); and it is sensitive to problem difficulty (Pinheiro‐Chagas et al. 2018). In the context of reading, this area is also involved in lexical decisions (Dien et al. 2013; Heim et al. 2009). Therefore, it is possible that activation here is associated with the recognition of meaningful symbolic information either in the form of arithmetic problems or known words.

Notably, we also found overlap in the right cerebellum for the adult group. While most of our knowledge about the neural substrates of high‐level cognition has been focused on cortical structures, recent evidence suggests that subcortical regions like the cerebellum also play an important role in multiple cognitive processes, including executive function, visuospatial processing, and others (Buckner 2013; Schmahmann 2019). Critically, there is evidence in the literature about studies finding significant activation in the cerebellum during phonological processing tasks (Baillieux et al. 2009; Feng et al. 2017; Linkersdörfer et al. 2012; Norton et al. 2014), as well as during arithmetic tasks (McDougle et al. 2022; Saban et al. 2024). One aspect of the functional characteristics of the cerebellum that might explain the convergence of both domains in this region has to do with its role in verbal working memory. Current models of verbal working memory have proposed the existence of cortical‐cerebellar networks that support different aspects of the phonological loop necessary to encode and retrieve verbal information (Baddeley 1992). More specifically, the inferior frontal gyrus, together with superior sections of the cerebellum, is thought to support an articulatory control system that makes possible the retrieval of verbal information during short‐term memory tasks (Chen and Desmond 2005b, 2005a; Hautzel et al. 2009). This evidence is in line with our findings of significant overlap in both the superior cerebellar areas and the inferior frontal gyrus. Even though our tasks were designed to tap into the retrieval of arithmetic facts and word sound structure stored in long‐term rather than short‐term memory, it is possible that similar phonological rehearsal processes have taken place during our experiment.

In addition to these regions, we also found evidence of overlap across domains along the middle and superior frontal gyrus only in the children sample. These regions are commonly associated with cognitive control processes, such as working memory, attentional regulation, and task monitoring (Duncan 2010; Fedorenko et al. 2013). Similar areas of the prefrontal cortex have also been linked to arithmetic, particularly in relation to strategy use and selection (Arsalidou et al. 2018; Delazer et al. 2005). Notably, the contribution of prefrontal regions to arithmetic tends to diminish with age, in parallel with an increased reliance on retrieval‐based strategies (Peters and De Smedt 2017; Rivera et al. 2005; Vogel and De Smedt 2021). Further research is needed to understand whether the role of these regions in verbal arithmetic follows a similar developmental trajectory as their role during word reading or more specifically phonological processing tasks.

### 4.2 Effect of Problem Size in the Overlap Between Arithmetic and Phonological Processing

Following our conjunction analysis, we examined whether the relationship between arithmetic and word reading differed for small vs. large problems along the clusters where overlap was observed. To address this question, we compared the activation for each condition along the clusters of overlap; but most importantly, we tested if there was a larger neural similarity between the distributed patterns of activation for rhyming and small problems, compared to rhyming and large problems. We hypothesized a stronger association between phonological processing and small problems, considering that the use of verbal strategies is typically more frequent during easier, well‐rehearsed problems. However, our results did not provide evidence supporting this prediction as we consistently found greater neural similarity between the rhyming and large problems, compared to rhyming and small problems. However, albeit different, both correlation values were significantly different from zero, suggesting that both arithmetic conditions recruited these regions in similar ways as the phonological processing task. In other words, our findings suggest that the overlap observed at the univariate level is not the result of functionally independent networks on similar anatomical areas, but rather similar mechanisms involved in both tasks.

The fact that we were unable to find a single cluster in which the neural patterns observed during the phonological processing task were more similar to small than large problems was unexpected. Our original prediction was based on the behavioral evidence suggesting that phonological processing abilities play a selective role supporting retrieval‐based arithmetic (Boets and De Smedt 2010; Matejko et al. 2022; Vukovic and Lesaux 2013), and the fact that retrieval strategies are more frequent for smaller, more familiar problems (Campbell and Xue 2001; De Smedt et al. 2011; LeFevre et al. 1996; Stanescu‐Cosson et al. 2000; Zbrodoff and Logan 2005). Nevertheless, it is possible that these results are not as contradictory as they may seem.

In the first place, the involvement of verbal mechanisms during larger problems involving procedural strategies is, by itself, not contradictory. The previous literature about retrieval versus computations has highlighted that problems that are typically retrieved from memory involve left‐lateralized language areas to a higher degree (i.e., left Angular Gyrus, Supramarginal Gyrus and Inferior Frontal Gyrus), whereas those solved using computations use parietal areas to a higher degree (Benn et al. 2012; Grabner et al. 2009; Lee 2000; Polspoel et al. 2017). These findings emphasize the *relative* differences across conditions, but do not mean that retrieval and, therefore, the activation of phonological representations for arithmetic does not happen at all during procedural strategies. For example, a problem like 8 + 7 could be solved by: (a) direct retrieval: “eight plus seven is fifteen”; (b) decomposition: 8 + 7 = 8 + 2 + 5 and then retrieval of “eight plus two is 10,” “ten plus five is fifteen”; (c) approximation to known facts: “eight plus eight is sixteen,” 16–1 = 15. In other words, while verbal retrieval mechanisms may be the key component of direct retrieval arithmetic, they may also have a supporting role during intermediate steps of procedural strategies. Ultimately, *small* and *large* problems are relative terms, not absolute categories that discriminate between qualitatively different arithmetic facts. Given the lack of direct strategy reports, we also need to consider the possibility that all the problems presented here were small or familiar enough that verbal strategies were always available to participants when responding to most of the trials, with minimal need for other procedures such as counting or decompositions of problems into smaller components. In addition, the small problems may have been so automatized that detecting a discrepancy between problem and solution in the context of our verification task may have been achieved with minimal involvement of phonological processing mechanisms.

The fact that the patterns of neural activity observed during small problems had, very consistently, the lowest similarity with the phonological processing condition may also be interpreted under an alternative hypothesis about the way small addition problems are solved. Some authors have proposed that small problems are not solved by verbal retrieval, but rather very fast counting (Baroody 1995; Barrouillet and Thevenot 2013; Uittenhove et al. 2016). This hypothesis has been sustained by behavioral data showing that even for very small addition problems that are solved very fast, response times increase monotonically with the magnitude of operands, suggesting the use of sequential counting procedures. However, recent neuroimaging evidence using multivariate analysis of EEG data has challenged this account (Grabner et al. 2022). The authors measured participants' brain activity during addition and multiplication and also collected trial‐specific strategy reports. If unlike multiplication, small addition problems are solved by means of highly automatized and fast counting procedures, this would translate into different neurophysiological responses that may be overlooked by behavioral paradigms or other slower neuroimaging techniques, but that are detectable at the high temporal resolution allowed by EEG measures. Their results, however, suggested similar rather than different neurophysiological responses during multiplication and small addition. Similarly, the results shown here suggest similar patterns for small problems and phonological processing, even if the similarity was lower when compared to the large problems. In other words, our analyses suggest that phonological processes were less prominent during small problems, but not completely absent.

Another alternative that we need to consider is that the overlapping effects observed here do not stem from shared specific mechanisms of verbal retrieval, but rather general cognitive control processes. In fact, some of the frontal and temporal regions where we found evidence of overlap have been associated with both a network of left‐lateralized frontal and temporal regions that supports linguistic processing (Fedorenko and Thompson‐Schill 2014), but also a more general multiple‐demand network that is involved in a vast range of cognitive processes (Assem et al. 2020; Duncan 2010; Duncan and Owen 2000). These networks include regions that are functionally independent despite being in very close anatomical proximity (Mineroff et al. 2018). These mechanisms of cognitive control are relatively consistent across different tasks, not only math and reading, and typically engage two main systems: a cingulo‐opercular network and a fronto‐parietal network (Dosenbach et al. 2007; Power and Petersen 2013). It is possible that at least some of the regions of significant overlap described here, especially those along the frontal cortex, result from the engagement of these mechanisms and, therefore, may not necessarily reflect specific links between phonology and arithmetic fact retrieval. For the arithmetic task, in particular, it is possible that the contrast of small + large > plus‐one may be tapping into regions sensitive to differences in difficulty between these conditions, therefore resulting in significant effects along general‐domain areas where activation is oftentimes modulated by difficulty or perceived effort (Braver et al. 1997; Dobryakova et al. 2017; Miri Ashtiani and Daliri 2023). However, for the reading task, both the letter matching and rhyming conditions were very similar in terms of the visual properties of the stimuli, the task instructions and the response selection requirements; but differed in a critical aspect: the control condition did not involve the access to phonological or semantic information.

What is more important, differences in task demands between experimental and control conditions could potentially explain some of the univariate results, but they do not necessarily explain why we also found significant similarities between the patterns for arithmetic and reading in these areas. In fact, several studies about this *multiple‐demand network* have shown that even though activation in these areas is sensitive to task demands across multiple domains, the multivariate patterns observed in these areas can be used to decode specific cognitive states or stimuli characteristics (Haynes and Rees 2006; Stiers et al. 2010; Thompson and Duncan 2009; Woolgar et al. 2011). Therefore, the multivariate analysis presented here further supports the idea that even if the overlap observed results from the role of general cognitive mechanisms, there seems to be a convergence across both domains in the way these mechanisms are recruited to subserve arithmetic problem solving and phonological decoding, which is an important factor explaining the relationship between these two abilities (Church 2023).

### 4.3 Limitations and Recommendations for Future Studies

Further research is needed to understand how the relationship between the neural mechanisms supporting reading and arithmetic is modulated by factors like problem size, operation types—most importantly—strategy use. In our study, for example, the lack of strategy reports represents a limitation as it is unclear to what extent the observed differences between small and large problems are the result of strategy or, more generally, problem size.

Building on the current findings, subsequent studies could investigate associations between arithmetic and reading in the context of different experimental paradigms. We used the data from a rhyming task that required individuals to evaluate whether the end of two words is the same or not. This task taps into the participant's ability to recognize and manipulate the sound structure of the words, an aspect of phonological abilities known as phonemic awareness (Liberman 1971, 1973). However, the research showing specific links between arithmetic—more specifically retrieval—and different subcomponents of reading suggests that other aspects like the rate of access to phonological representations may have a critical role in this relationship (see Araújo et al. 2015 for a meta‐analysis), as evidenced by multiple studies showing significant associations between rapid automatized naming skills and arithmetic performance (Cui et al. 2017; Donker et al. 2016; Escobar et al. 2021; Koponen et al. 2016).

## 5 Conclusions

A longstanding hypothesis about specific mechanisms supporting the observed associations between mathematics and reading poses that the ability to recall arithmetic facts from long‐term memory is supported by the same neural network involved in verbal retrieval that supports phonological processing. However, neuroimaging evidence directly testing this hypothesis remains to be very scarce. Our study tested this hypothesis by examining the neural correlates of arithmetic and phonological processing within participants. We demonstrated that word rhyming and arithmetic recruit spatially overlapping brain regions along the canonical language network, including areas of the frontal and temporal cortex. Moreover, using multivariate analyses we provided further evidence that both processes elicit similar distributed patterns of brain activation in the areas where overlap was identified. Contrary to our expectation, greater similarity was observed between the activation patterns corresponding to phonological processing and large problems, compared to small problems. Our findings suggest the need for further research regarding the role of problem size and strategy use in the recruitment of these verbal mechanisms during arithmetic problem solving. In addition, the convergence of arithmetic and phonological processing along similar brain regions was observed for both adults and children, suggesting that this association is important across multiple developmental stages.

## Funding

This work was supported by the Natural Sciences and Engineering Research Council of Canada (342192‐RGPIN), Jacobs Foundation (2017‐1261‐01), and Canada Research Chairs (950‐232363).

## Conflicts of Interest

The authors declare no conflicts of interest.

## Supporting information


**Data S1:** hbm70446‐sup‐0001‐Supinfo.docx.

## Data Availability

The data that support the findings of this study are available from the corresponding author upon reasonable request.

## References

[hbm70446-bib-0001] Amland, T. , A. Lervåg , and M. Melby‐Lervåg . 2021. “Comorbidity Between Math and Reading Problems: Is Phonological Processing a Mutual Factor?” Frontiers in Human Neuroscience 14: 577304. 10.3389/fnhum.2020.577304. 33488369 PMC7817538

[hbm70446-bib-0002] Andin, J. , P. Fransson , J. Rönnberg , and M. Rudner . 2015. “Phonology and Arithmetic in the Language–Calculation Network.” Brain and Language 143: 97–105. 10.1016/j.bandl.2015.02.004. 25797099

[hbm70446-bib-0003] Araújo, S. , A. Reis , K. M. Petersson , and L. Faísca . 2015. “Rapid Automatized Naming and Reading Performance: A Meta‐Analysis.” Journal of Educational Psychology 107, no. 3: 868–883. 10.1037/edu0000006.

[hbm70446-bib-0004] Arsalidou, M. , M. Pawliw‐Levac , M. Sadeghi , and J. Pascual‐Leone . 2018. “Brain Areas Associated With Numbers and Calculations in Children: Meta‐Analyses of fMRI Studies.” Developmental Cognitive Neuroscience 30: 239–250. 10.1016/j.dcn.2017.08.002. 28844728 PMC6969084

[hbm70446-bib-0005] Assem, M. , M. F. Glasser , D. C. Van Essen , and J. Duncan . 2020. “A Domain‐General Cognitive Core Defined in Multimodally Parcellated Human Cortex.” Cerebral Cortex 30, no. 8: 4361–4380. 10.1093/cercor/bhaa023. 32244253 PMC7325801

[hbm70446-bib-0006] Baddeley, A. 1992. “Working Memory.” Science 255, no. 5044: 556–559. 10.1126/science.1736359. 1736359

[hbm70446-bib-0007] Baillieux, H. , E. J. M. Vandervliet , M. Manto , P. M. Parizel , P. P. D. Deyn , and P. Mariën . 2009. “Developmental Dyslexia and Widespread Activation Across the Cerebellar Hemispheres.” Brain and Language 108, no. 2: 122–132. 10.1016/j.bandl.2008.10.001. 18986695

[hbm70446-bib-0008] Baroody, A. J. 1995. “The Role of the Number‐After Rule in the Invention of Computational Shortcuts.” Cognition and Instruction 13, no. 2: 189–219. 10.1207/s1532690xci1302_2.

[hbm70446-bib-0009] Barrouillet, P. , M. Mignon , and C. Thevenot . 2008. “Strategies in Subtraction Problem Solving in Children.” Journal of Experimental Child Psychology 99, no. 4: 233–251. 10.1016/j.jecp.2007.12.001. 18241880

[hbm70446-bib-0010] Barrouillet, P. , and C. Thevenot . 2013. “On the Problem‐Size Effect in Small Additions: Can We Really Discard Any Counting‐Based Account?” Cognition 128, no. 1: 35–44. 10.1016/j.cognition.2013.02.018. 23583543

[hbm70446-bib-0011] Benn, Y. , Y. Zheng , I. D. Wilkinson , M. Siegal , and R. Varley . 2012. “Language in Calculation: A Core Mechanism?” Neuropsychologia 50, no. 1: 1–10. 10.1016/j.neuropsychologia.2011.09.045. 22079204

[hbm70446-bib-0012] Boets, B. , and B. De Smedt . 2010. “Single‐Digit Arithmetic in Children With Dyslexia.” Dyslexia 16: 183–191. 10.1002/dys.403. 20440746

[hbm70446-bib-0013] Bolger, D. J. , C. A. Perfetti , and W. Schneider . 2005. “Cross‐Cultural Effect on the Brain Revisited: Universal Structures Plus Writing System Variation.” Human Brain Mapping 25, no. 1: 92–104. 10.1002/hbm.20124. 15846818 PMC6871743

[hbm70446-bib-0014] Brady, S. A. , and D. P. Shankweiler . 2013. Phonological Processes in Literacy: A Tribute to Isabelle Y. Liberman. Routledge.

[hbm70446-bib-0015] Braver, T. S. , J. D. Cohen , L. E. Nystrom , J. Jonides , E. E. Smith , and D. C. Noll . 1997. “A Parametric Study of Prefrontal Cortex Involvement in Human Working Memory.” NeuroImage 5, no. 1: 49–62. 10.1006/nimg.1996.0247. 9038284

[hbm70446-bib-0016] Brown, R. 2021. “Member's Paper: Driving Longevity Through Educational Attainment—A Literature Review.”

[hbm70446-bib-0017] Brozdowski, C. , and J. Booth . 2021. “Reading Skill Correlates in Frontal Cortex During Semantic and Phonological Processing.” OSF. 10.31234/osf.io/d3mj7.

[hbm70446-bib-0018] Buckner, R. L. 2013. “The Cerebellum and Cognitive Function: 25 Years of Insight From Anatomy and Neuroimaging.” Neuron 80, no. 3: 807–815. 10.1016/j.neuron.2013.10.044. 24183029

[hbm70446-bib-0019] Cai, M. B. , N. W. Schuck , J. W. Pillow , and Y. Niv . 2019. “Representational Structure or Task Structure? Bias in Neural Representational Similarity Analysis and a Bayesian Method for Reducing Bias.” PLoS Computational Biology 15, no. 5: e1006299. 10.1371/journal.pcbi.1006299. 31125335 PMC6553797

[hbm70446-bib-0020] Campbell, J. , and Q. Xue . 2001. “Cognitive Arithmetic Across Cultures.” Journal of Experimental Psychology: General 130, no. 2: 299–315. 10.1037/0096-3445.130.2.299. 11409105

[hbm70446-bib-0021] Cao, F. , T. Bitan , and J. R. Booth . 2008. “Effective Brain Connectivity in Children With Reading Difficulties During Phonological Processing.” Brain and Language 107, no. 2: 91–101. 10.1016/j.bandl.2007.12.009. 18226833 PMC2676797

[hbm70446-bib-0022] Chen, S. H. A. , and J. E. Desmond . 2005a. “Cerebrocerebellar Networks During Articulatory Rehearsal and Verbal Working Memory Tasks.” NeuroImage 24, no. 2: 332–338. 10.1016/j.neuroimage.2004.08.032. 15627576

[hbm70446-bib-0023] Chen, S. H. A. , and J. E. Desmond . 2005b. “Temporal Dynamics of Cerebro‐Cerebellar Network Recruitment During a Cognitive Task.” Neuropsychologia 43, no. 9: 1227–1237. 10.1016/j.neuropsychologia.2004.12.015. 15949507

[hbm70446-bib-0024] Child, A. E. , P. T. Cirino , J. M. Fletcher , E. G. Willcutt , and L. S. Fuchs . 2019. “A Cognitive Dimensional Approach to Understanding Shared and Unique Contributions to Reading, Math, and Attention Skills.” Journal of Learning Disabilities 52, no. 1: 15–30. 10.1177/0022219418775115. 29779434 PMC6212329

[hbm70446-bib-0025] Church, J. A. 2023. “The Brain's Control Networks in Reading: Insights From Cross‐Task Studies of Youth.” Mind, Brain, and Education 17, no. 4: 257–266. 10.1111/mbe.12372. PMC11091959 38745918

[hbm70446-bib-0026] Clercq‐Quaegebeur, M. D. , S. Casalis , B. Vilette , M.‐P. Lemaitre , and L. Vallée . 2018. “Arithmetic Abilities in Children With Developmental Dyslexia: Performance on French ZAREKI‐R Test.” Journal of Learning Disabilities 51, no. 3: 236–249. 10.1177/0022219417690355. 28134569

[hbm70446-bib-0027] Compton, D. L. 2003. “Modeling the Relationship Between Growth in Rapid Naming Speed and Growth in Decoding Skill in First‐Grade Children.” Journal of Educational Psychology 95, no. 2: 225–239. 10.1037/0022-0663.95.2.225.

[hbm70446-bib-0163] Cox, R. W. , G. Chen , D. R. Glen , R. C. Reynolds , and P. A. Taylor . 2017. “fMRI Clustering and False‐Positive Rates.” Proceedings of the National Academy of Sciences 114, no. 17. 10.1073/pnas.1614961114. PMC5410825 28420798

[hbm70446-bib-0028] Cui, J. , G. K. Georgiou , Y. Zhang , Y. Li , H. Shu , and X. Zhou . 2017. “Examining the Relationship Between Rapid Automatized Naming and Arithmetic Fluency in Chinese Kindergarten Children.” Journal of Experimental Child Psychology 154: 146–163. 10.1016/j.jecp.2016.10.008. 27883911

[hbm70446-bib-0029] De Smedt, B. 2018. “Chapter 3—Language and Arithmetic: The Potential Role of Phonological Processing.” In Heterogeneity of Function in Numerical Cognition, edited by A. Henik and W. Fias , 51–74. Academic Press. 10.1016/B978-0-12-811529-9.00003-0.

[hbm70446-bib-0030] De Smedt, B. , and B. Boets . 2010. “Phonological Processing and Arithmetic Fact Retrieval: Evidence From Developmental Dyslexia.” Neuropsychologia 48, no. 14: 3973–3981. 10.1016/j.neuropsychologia.2010.10.018. 20965205

[hbm70446-bib-0031] De Smedt, B. , I. D. Holloway , and D. Ansari . 2011. “Effects of Problem Size and Arithmetic Operation on Brain Activation During Calculation in Children With Varying Levels of Arithmetical Fluency.” NeuroImage 57, no. 3: 771–781. 10.1016/j.neuroimage.2010.12.037. 21182966

[hbm70446-bib-0032] De Smedt, B. , J. Taylor , L. Archibald , and D. Ansari . 2010. “How Is Phonological Processing Related to Individual Differences in Children's Arithmetic Skills?” Developmental Science 13, no. 3: 508–520. 10.1111/j.1467-7687.2009.00897.x. 20443971

[hbm70446-bib-0033] Dehaene, S. 1992. “Varieties of Numerical Abilities.” Cognition 44, no. 1: 1–42. 10.1016/0010-0277(92)90049-N. 1511583

[hbm70446-bib-0034] Dehaene, S. , and L. Cohen . 1995. “Towards an Anatomical and Functional Model of Number Processing.” Mathematical Cognition 1: 83–120.

[hbm70446-bib-0035] Dehaene, S. , and L. Cohen . 1998. “Levels of Representation in Number Processing.” In Handbook of Neurolinguistics, 331–341. Elsevier. 10.1016/B978-012666055-5/50026-5.

[hbm70446-bib-0036] Dehaene, S. , N. Molko , L. Cohen , and A. J. Wilson . 2004. “Arithmetic and the Brain.” Current Opinion in Neurobiology 14, no. 2: 218–224. 10.1016/j.conb.2004.03.008. 15082328

[hbm70446-bib-0037] Delazer, M. , A. Ischebeck , F. Domahs , et al. 2005. “Learning by Strategies and Learning by Drill—Evidence From an fMRI Study.” NeuroImage 25, no. 3: 838–849. 10.1016/j.neuroimage.2004.12.009. 15808984

[hbm70446-bib-0038] Diedrichsen, J. , E. Berlot , M. Mur , H. H. Schütt , M. Shahbazi , and N. Kriegeskorte . 2021. “Comparing Representational Geometries Using Whitened Unbiased‐Distance‐Matrix Similarity.” *In* arXiv [stat. AP]. http://arxiv.org/abs/2007.02789.

[hbm70446-bib-0039] Dien, J. , E. S. Brian , D. L. Molfese , and B. T. Gold . 2013. “Combined ERP/fMRI Evidence for Early Word Recognition Effects in the Posterior Inferior Temporal Gyrus.” Cortex 49, no. 9: 2307–2321. 10.1016/j.cortex.2013.03.008. 23701693 PMC3758432

[hbm70446-bib-0040] Ding, H. , and M. Homer . 2020. “Interpreting Mathematics Performance in PISA: Taking Account of Reading Performance.” International Journal of Educational Research 102: 101566. 10.1016/j.ijer.2020.101566.

[hbm70446-bib-0041] Dirks, E. , G. Spyer , E. C. D. M. van Lieshout , and L. de Sonneville . 2008. “Prevalence of Combined Reading and Arithmetic Disabilities.” Journal of Learning Disabilities 41, no. 5: 460–473. 10.1177/0022219408321128. 18768777

[hbm70446-bib-0042] Dobryakova, E. , R. K. Jessup , and E. Tricomi . 2017. “Modulation of Ventral Striatal Activity by Cognitive Effort.” NeuroImage 147: 330–338. 10.1016/j.neuroimage.2016.12.029. 27989778 PMC5303632

[hbm70446-bib-0043] Donker, M. , E. Kroesbergen , E. Slot , S. Van Viersen , and E. De Bree . 2016. “Alphanumeric and Non‐Alphanumeric Rapid Automatized Naming in Children With Reading and/or Spelling Difficulties and Mathematical Difficulties.” Learning and Individual Differences 47: 80–87. 10.1016/j.lindif.2015.12.011.

[hbm70446-bib-0044] Dosenbach, N. U. F. , D. A. Fair , F. M. Miezin , et al. 2007. “Distinct Brain Networks for Adaptive and Stable Task Control in Humans.” Proceedings of the National Academy of Sciences 104, no. 26: 11073–11078. 10.1073/pnas.0704320104. PMC1904171 17576922

[hbm70446-bib-0045] Duncan, J. 2010. “The Multiple‐Demand (MD) System of the Primate Brain: Mental Programs for Intelligent Behaviour.” Trends in Cognitive Sciences 14, no. 4: 172–179. 10.1016/j.tics.2010.01.004. 20171926

[hbm70446-bib-0046] Duncan, J. , and A. M. Owen . 2000. “Common Regions of the Human Frontal Lobe Recruited by Diverse Cognitive Demands.” Trends in Neurosciences 23, no. 10: 475–483. 10.1016/S0166-2236(00)01633-7. 11006464

[hbm70446-bib-0047] Escobar, J.‐P. , F. Porflitt , and F. Ceric . 2021. “Evaluating the Rapid Automatized Naming and Arithmetical Fluency Relationship in Chilean First Grade Students.” Educational Psychology 41, no. 6: 730–747. 10.1080/01443410.2021.1900545.

[hbm70446-bib-0162] Esteban, O. , C. J. Markiewicz , R. W. Blair , et al. 2018. “fMRIPrep: A Robust Preprocessing Pipeline for Functional MRI.” Nature Methods 16, no. 1: 111–116. 10.1038/s41592-018-0235-4. 30532080 PMC6319393

[hbm70446-bib-0048] Evans, T. M. , D. L. Flowers , M. M. Luetje , E. Napoliello , and G. F. Eden . 2016. “Functional Neuroanatomy of Arithmetic and Word Reading and Its Relationship to Age.” NeuroImage 143: 304–315. 10.1016/j.neuroimage.2016.08.048. 27566261 PMC5124535

[hbm70446-bib-0049] Fedorenko, E. , M. K. Behr , and N. Kanwisher . 2011. “Functional Specificity for High‐Level Linguistic Processing in the Human Brain.” Proceedings of the National Academy of Sciences 108, no. 39: 16428–16433. 10.1073/pnas.1112937108. PMC3182706 21885736

[hbm70446-bib-0050] Fedorenko, E. , J. Duncan , and N. Kanwisher . 2012. “Language‐Selective and Domain‐General Regions Lie Side by Side Within Broca's Area.” Current Biology 22, no. 21: 2059–2062. 10.1016/j.cub.2012.09.011. 23063434 PMC3494832

[hbm70446-bib-0051] Fedorenko, E. , J. Duncan , and N. Kanwisher . 2013. “Broad Domain Generality in Focal Regions of Frontal and Parietal Cortex.” Proceedings of the National Academy of Sciences 110, no. 41: 16616–16621. 10.1073/pnas.1315235110. PMC3799302 24062451

[hbm70446-bib-0052] Fedorenko, E. , and S. L. Thompson‐Schill . 2014. “Reworking the Language Network.” Trends in Cognitive Sciences 18, no. 3: 120–126. 10.1016/j.tics.2013.12.006. 24440115 PMC4091770

[hbm70446-bib-0053] Feng, X. , L. Li , M. Zhang , et al. 2017. “Dyslexic Children Show Atypical Cerebellar Activation and Cerebro‐Cerebellar Functional Connectivity in Orthographic and Phonological Processing.” Cerebellum 16, no. 2: 496–507. 10.1007/s12311-016-0829-2. 27785760

[hbm70446-bib-0165] Friston, K. J. , J. Ashburner , S. J. Kiebel , T. E. Nichols , and W. D. Penny . 2007. Statistical Parametric Mapping Statistical Parametric Mapping. The Analysis of Functional Brain Images. Academic Press. 10.1016/b978-0-12-372560-8.x5000-1.

[hbm70446-bib-0055] Fuchs, L. S. , D. L. Compton , D. Fuchs , K. Paulsen , J. D. Bryant , and C. L. Hamlett . 2005. “The Prevention, Identification, and Cognitive Determinants of Math Difficulty.” Journal of Educational Psychology 97, no. 3: 493–513. 10.1037/0022-0663.97.3.493.

[hbm70446-bib-0056] Fuchs, L. S. , D. Fuchs , D. L. Compton , et al. 2006. “The Cognitive Correlates of Third‐Grade Skill in Arithmetic, Algorithmic Computation, and Arithmetic Word Problems.” Journal of Educational Psychology 98, no. 1: 29–43. 10.1037/0022-0663.98.1.29.

[hbm70446-bib-0057] Geary, D. C. , C. C. Bow‐Thomas , F. Liu , and R. S. Siegler . 1996. “Development of Arithmetical Competencies in Chinese and American Children: Influence of Age, Language, and Schooling.” Child Development 67, no. 5: 2022–2044. 9022227

[hbm70446-bib-0058] Geary, D. C. , M. K. Hoard , L. Nugent , Z. E. Ünal , and J. E. Scofield . 2020. “Comorbid Learning Difficulties in Reading and Mathematics: The Role of Intelligence and In‐Class Attentive Behavior.” Frontiers in Psychology 11: 572099. 10.3389/fpsyg.2020.572099. 33312148 PMC7701335

[hbm70446-bib-0059] Gilmore, C. K. , S. E. McCarthy , and E. S. Spelke . 2007. “Symbolic Arithmetic Knowledge Without Instruction.” Nature 447, no. 7144: 589–591. 10.1038/nature05850. 17538620

[hbm70446-bib-0060] Göbel, S. M. , S. E. Watson , A. Lervåg , and C. Hulme . 2014. “Children's Arithmetic Development: It Is Number Knowledge, Not the Approximate Number Sense, That Counts.” Psychological Science 25, no. 3: 789–798. 10.1177/0956797613516471. 24482406

[hbm70446-bib-0061] Grabner, R. H. , D. Ansari , K. Koschutnig , G. Reishofer , F. Ebner , and C. Neuper . 2009. “To Retrieve or to Calculate? Left Angular Gyrus Mediates the Retrieval of Arithmetic Facts During Problem Solving.” Neuropsychologia 47, no. 2: 604–608. 10.1016/j.neuropsychologia.2008.10.013. 19007800

[hbm70446-bib-0062] Grabner, R. H. , C. Brunner , V. Lorenz , S. E. Vogel , and B. De Smedt . 2022. “Fact Retrieval or Compacted Counting in Arithmetic—A Neurophysiological Investigation of Two Hypotheses.” Journal of Experimental Psychology: Learning, Memory, and Cognition 48, no. 2: 199–212. 10.1037/xlm0000982. 33539170

[hbm70446-bib-0063] Graves, W. W. , J. Purcell , D. Rothlein , D. J. Bolger , M. Rosenberg‐Lee , and R. Staples . 2023. “Correspondence Between Cognitive and Neural Representations for Phonology, Orthography, and Semantics in Supramarginal Compared to Angular Gyrus.” Brain Structure and Function 228, no. 1: 255–271. 10.1007/s00429-022-02590-y. 36326934

[hbm70446-bib-0064] Greiner de Magalhães, C. , C. B. Mervis , and C. Cardoso‐Martins . 2021. “Cognitive Predictors of Arithmetic, Reading, and Spelling in Brazilian Portuguese‐Speaking Children.” Reading and Writing 34, no. 1: 171–198. 10.1007/s11145-020-10062-0.

[hbm70446-bib-0065] Grossi, G. , D. Coch , S. Coffey‐Corina , P. J. Holcomb , and H. J. Neville . 2001. “Phonological Processing in Visual Rhyming: A Developmental ERP Study.” Journal of Cognitive Neuroscience 13, no. 5: 610–625. 10.1162/089892901750363190. 11506660

[hbm70446-bib-0066] Grotheer, M. , B. Jeska , and K. Grill‐Spector . 2018. “A Preference for Mathematical Processing Outweighs the Selectivity for Arabic Numbers in the Inferior Temporal Gyrus.” NeuroImage 175: 188–200. 10.1016/j.neuroimage.2018.03.064. 29604456 PMC6173953

[hbm70446-bib-0067] Hautzel, H. , F. M. Mottaghy , K. Specht , H.‐W. Müller , and B. J. Krause . 2009. “Evidence of a Modality‐Dependent Role of the Cerebellum in Working Memory? An fMRI Study Comparing Verbal and Abstract n‐Back Tasks.” NeuroImage 47, no. 4: 2073–2082. 10.1016/j.neuroimage.2009.06.005. 19524048

[hbm70446-bib-0068] Haxby, J. V. , M. I. Gobbini , M. L. Furey , A. Ishai , J. L. Schouten , and P. Pietrini . 2001. “Distributed and Overlapping Representations of Faces and Objects in Ventral Temporal Cortex.” Science 293, no. 5539: 2425–2430. 10.1126/science.1063736. 11577229

[hbm70446-bib-0069] Haynes, J.‐D. , and G. Rees . 2006. “Decoding Mental States From Brain Activity in Humans.” Nature Reviews Neuroscience 7, no. 7: 523–534. 10.1038/nrn1931. 16791142

[hbm70446-bib-0070] Heim, S. , S. B. Eickhoff , A. K. Ischebeck , A. D. Friederici , K. E. Stephan , and K. Amunts . 2009. “Effective Connectivity of the Left BA 44, BA 45, and Inferior Temporal Gyrus During Lexical and Phonological Decisions Identified With DCM.” Human Brain Mapping 30, no. 2: 392–402. 10.1002/hbm.20512. 18095285 PMC6870893

[hbm70446-bib-0071] Henrich, J. , S. J. Heine , and A. Norenzayan . 2010. “The Weirdest People in the World?” Behavioral and Brain Sciences 33, no. 2–3: 61–83. 10.1017/S0140525X0999152X. 20550733

[hbm70446-bib-0072] Hermes, D. , V. Rangarajan , B. L. Foster , et al. 2017. “Electrophysiological Responses in the Ventral Temporal Cortex During Reading of Numerals and Calculation.” Cerebral Cortex 27, no. 1: 567–575. 10.1093/cercor/bhv250. 26503267 PMC5939218

[hbm70446-bib-0073] Hetch, S. A. , J. K. Torgensen , R. K. Wagner , and C. A. Rashotte . 2001. “The Relations Between Phonological Processing Abilities and Emerging Individual Differences in Mathematical Computation Skills: A Longitudinal Study From Second to Fifth Grades.” Journal of Experimental Child Psychology 79: 192–227. 10.1006/jecp.2000.2586. 11343408

[hbm70446-bib-0074] Hoeft, F. , A. Meyler , A. Hernandez , et al. 2007. “Functional and Morphometric Brain Dissociation Between Dyslexia and Reading Ability.” Proceedings of the National Academy of Sciences 104, no. 10: 4234–4239. 10.1073/pnas.0609399104. PMC1820738 17360506

[hbm70446-bib-0075] Hulme, C. , and M. J. Snowling . 2013. “Learning to Read: What we Know and What we Need to Understand Better.” Child Development Perspectives 7, no. 1: 1–5. 10.1111/cdep.12005. PMC4538787 26290678

[hbm70446-bib-0076] Imbo, I. , and A. Vandierendonck . 2008. “Practice Effects on Strategy Selection and Strategy Efficiency in Simple Mental Arithmetic.” Psychological Research 72, no. 5: 528–541. 10.1007/s00426-007-0128-0. 17906877

[hbm70446-bib-0077] Ischebeck, A. , L. Zamarian , M. Schocke , and M. Delazer . 2009. “Flexible Transfer of Knowledge in Mental Arithmetic—An fMRI Study.” NeuroImage 44, no. 3: 1103–1112. 10.1016/j.neuroimage.2008.10.025. 19027075

[hbm70446-bib-0078] Jöbstl, V. , A. F. Steiner , P. Deimann , U. Kastner‐Koller , and K. Landerl . 2023. “A‐B‐3—Associations and Dissociations of Reading and Arithmetic: Is Domain‐Specific Prediction Outdated?” PLoS One 18, no. 5: e0285437. 10.1371/journal.pone.0285437. 37172049 PMC10180600

[hbm70446-bib-0079] Joyner, R. E. , and R. K. Wagner . 2020. “Co‐Occurrence of Reading Disabilities and Math Disabilities: A Meta‐Analysis.” Scientific Studies of Reading 24, no. 1: 14–22. 10.1080/10888438.2019.1593420. 32051676 PMC7015531

[hbm70446-bib-0080] Katzev, M. , O. Tüscher , J. Hennig , C. Weiller , and C. P. Kaller . 2013. “Revisiting the Functional Specialization of Left Inferior Frontal Gyrus in Phonological and Semantic Fluency: The Crucial Role of Task Demands and Individual Ability.” Journal of Neuroscience: The Official Journal of the Society for Neuroscience 33, no. 18: 7837–7845. 10.1523/JNEUROSCI.3147-12.2013. 23637175 PMC6618954

[hbm70446-bib-0081] Koponen, T. , P. Salmi , M. Torppa , et al. 2016. “Counting and Rapid Naming Predict the Fluency of Arithmetic and Reading Skills.” Contemporary Educational Psychology 44: 83–94. 10.1016/j.cedpsych.2016.02.004.

[hbm70446-bib-0082] Korhonen, J. , K. Linnanmäki , and P. Aunio . 2012. “Language and Mathematical Performance: A Comparison of Lower Secondary School Students With Different Level of Mathematical Skills.” Scandinavian Journal of Educational Research 56, no. 3: 333–344. 10.1080/00313831.2011.599423.

[hbm70446-bib-0083] Korpershoek, H. , H. Kuyper , and G. van der Werf . 2015. “The Relation Between Students' Math and Reading Ability and Their Mathematics, Physics, and Chemistry Examination Grades in Secondary Education.” International Journal of Science and Mathematics Education 13, no. 5: 1013–1037. 10.1007/s10763-014-9534-0.

[hbm70446-bib-0084] Korpipää, H. , T. Koponen , M. Aro , et al. 2017. “Covariation Between Reading and Arithmetic Skills From Grade 1 to Grade 7.” Contemporary Educational Psychology 51: 131–140. 10.1016/j.cedpsych.2017.06.005.

[hbm70446-bib-0085] Kriegeskorte, N. 2008. “Representational Similarity Analysis—Connecting the Branches of Systems Neuroscience.” Frontiers in Systems Neuroscience 2: 249. 10.3389/neuro.06.004.2008. PMC2605405 19104670

[hbm70446-bib-0086] Kriegeskorte, N. , and J. Diedrichsen . 2019. “Peeling the Onion of Brain Representations.” Annual Review of Neuroscience 42, no. 1: 407–432. 10.1146/annurev-neuro-080317-061906. 31283895

[hbm70446-bib-0087] Kriegeskorte, N. , and P. K. Douglas . 2019. “Interpreting Encoding and Decoding Models.” Current Opinion in Neurobiology 55: 167–179. 10.1016/j.conb.2019.04.002. 31039527 PMC6705607

[hbm70446-bib-0088] Kriegeskorte, N. , R. Goebel , and P. Bandettini . 2006. “Information‐Based Functional Brain Mapping.” Proceedings of the National Academy of Sciences 103, no. 10: 3863–3868. 10.1073/pnas.0600244103. PMC1383651 16537458

[hbm70446-bib-0089] Landerl, K. , A. Castles , and R. Parrila . 2022. “Cognitive Precursors of Reading: A Cross‐Linguistic Perspective.” Scientific Studies of Reading 26, no. 2: 111–124. 10.1080/10888438.2021.1983820.

[hbm70446-bib-0090] Landerl, K. , B. Fussenegger , K. Moll , and E. Willburger . 2009. “Dyslexia and Dyscalculia: Two Learning Disorders With Different Cognitive Profiles.” Journal of Experimental Child Psychology 103, no. 3: 309–324. 10.1016/j.jecp.2009.03.006. 19398112

[hbm70446-bib-0091] Landerl, K. , and K. Moll . 2010. “Comorbidity of Learning Disorders: Prevalence and Familial Transmission.” Journal of Child Psychology and Psychiatry 51, no. 3: 287–294. 10.1111/j.1469-7610.2009.02164.x. 19788550

[hbm70446-bib-0092] Lee, K.‐M. 2000. “Cortical Areas Differentially Involved in Multiplication and Subtraction: A Functional Magnetic Resonance Imaging Study and Correlation With a Case of Selective Acalculia.” Annals of Neurology 48, no. 4: 657–661. 10.1002/1531-8249(200010)48:4<657::AID-ANA13>3.0.CO;2-K. 11026450

[hbm70446-bib-0093] LeFevre, J.‐A. , G. S. Sadesky , and J. Bisanz . 1996. “Selection of Procedures in Mental Addition: Reassessing the Problem Size Effect in Adults.” Journal of Experimental Psychology: Learning, Memory, and Cognition 22, no. 1: 216–230. 10.1037/0278-7393.22.1.216.

[hbm70446-bib-0094] Liberman, I. Y. 1971. “Basic Research in Speech and Lateralization of Language: Some Implications for Reading Disability.” Bulletin of the Orton Society 21, no. 1: 71–87. 10.1007/BF02663712.

[hbm70446-bib-0095] Liberman, I. Y. 1973. “Segmentation of the Spoken Word and Reading Acquisition.” Bulletin of the Orton Society 23: 65–77.

[hbm70446-bib-0096] Linkersdörfer, J. , J. Lonnemann , S. Lindberg , M. Hasselhorn , and C. J. Fiebach . 2012. “Grey Matter Alterations co‐Localize With Functional Abnormalities in Developmental Dyslexia: An ALE Meta‐Analysis.” PLoS One 7, no. 8: e43122. 10.1371/journal.pone.0043122. 22916214 PMC3423424

[hbm70446-bib-0097] Lytle, M. N. , T. Bitan , and J. R. Booth . 2020. “A Neuroimaging Dataset on Orthographic, Phonological and Semantic Word Processing in School‐Aged Children.” Data in Brief 28: 105091. 10.1016/j.dib.2019.105091. 31956678 PMC6957861

[hbm70446-bib-0098] Martin, A. , M. Schurz , M. Kronbichler , and F. Richlan . 2015. “Reading in the Brain of Children and Adults: A Meta‐Analysis of 40 Functional Magnetic Resonance Imaging Studies.” Human Brain Mapping 36, no. 5: 1963–1981. 10.1002/hbm.22749. 25628041 PMC4950303

[hbm70446-bib-0099] Matejko, A. A. , and D. Ansari . 2017. “How Do Individual Differences in Children's Domain Specific and Domain General Abilities Relate to Brain Activity Within the Intraparietal Sulcus During Arithmetic? An fMRI Study.” Human Brain Mapping 38, no. 8: 3941–3956. 10.1002/hbm.23640. 28488352 PMC6866897

[hbm70446-bib-0100] Matejko, A. A. , and D. Ansari . 2019. “The Neural Association Between Arithmetic and Basic Numerical Processing Depends on Arithmetic Problem Size and Not Chronological Age.” Developmental Cognitive Neuroscience 37: 100653. 10.1016/j.dcn.2019.100653. 31102959 PMC6969316

[hbm70446-bib-0101] Matejko, A. A. , and D. Ansari . 2021. “Shared Neural Circuits for Visuospatial Working Memory and Arithmetic in Children and Adults.” Journal of Cognitive Neuroscience 33, no. 6: 1003–1019. 10.1162/jocn_a_01695. 34428783

[hbm70446-bib-0102] Matejko, A. A. , M. Lozano , N. Schlosberg , et al. 2022. “The Relationship Between Phonological Processing and Arithmetic in Children With Learning Disabilities.” Developmental Science 26, no. 2: e13294. 10.1111/desc.13294. 35727164 PMC9768103

[hbm70446-bib-0103] McDougle, S. D. , J. S. Tsay , B. Pitt , et al. 2022. “Continuous Manipulation of Mental Representations Is Compromised in Cerebellar Degeneration.” Brain 145, no. 12: 4246–4263. 10.1093/brain/awac072. 35202465 PMC10200308

[hbm70446-bib-0104] McLaughlin, M. J. , K. E. Speirs , and E. D. Shenassa . 2014. “Reading Disability and Adult Attained Education and Income: Evidence From a 30‐Year Longitudinal Study of a Population‐Based Sample.” Journal of Learning Disabilities 47, no. 4: 374–386. 10.1177/0022219412458323. 22983608

[hbm70446-bib-0105] Merkley, R. , B. Conrad , G. Price , and D. Ansari . 2019. “Investigating the Visual Number Form Area: A Replication Study.” Royal Society Open Science 6, no. 10: 182067. 10.1098/rsos.182067. 31824678 PMC6837224

[hbm70446-bib-0106] Mineroff, Z. , I. A. Blank , K. Mahowald , and E. Fedorenko . 2018. “A Robust Dissociation Among the Language, Multiple Demand, and Default Mode Networks: Evidence From Inter‐Region Correlations in Effect Size.” Neuropsychologia 119: 501–511. 10.1016/j.neuropsychologia.2018.09.011. 30243926 PMC6191329

[hbm70446-bib-0107] Miri Ashtiani, S. N. , and M. R. Daliri . 2023. “Identification of Cognitive Load‐Dependent Activation Patterns Using Working Memory Task‐Based fMRI at Various Levels of Difficulty.” Scientific Reports 13, no. 1: 16476. 10.1038/s41598-023-43837-w. 37777667 PMC10543376

[hbm70446-bib-0108] Nili, H. , A. Walther , A. Alink , and N. Kriegeskorte . 2020. “Inferring Exemplar Discriminability in Brain Representations.” PLoS One 15, no. 6: e0232551. 10.1371/journal.pone.0232551. 32520962 PMC7286518

[hbm70446-bib-0109] Norton, E. S. , J. M. Black , L. M. Stanley , et al. 2014. “Functional Neuroanatomical Evidence for the Double‐Deficit Hypothesis of Developmental Dyslexia.” Neuropsychologia 61: 235–246. 10.1016/j.neuropsychologia.2014.06.015. 24953957 PMC4339699

[hbm70446-bib-0110] Östergren, R. , and U. Träff . 2013. “Early Number Knowledge and Cognitive Ability Affect Early Arithmetic Ability.” Journal of Experimental Child Psychology 115, no. 3: 405–421. 10.1016/j.jecp.2013.03.007. 23665177

[hbm70446-bib-0111] Passolunghi, M. C. , B. Vercelloni , and H. Schadee . 2007. “The Precursors of Mathematics Learning: Working Memory, Phonological Ability and Numerical Competence.” Cognitive Development 22, no. 2: 165–184. 10.1016/j.cogdev.2006.09.001.

[hbm70446-bib-0112] Peelen, M. V. , and P. E. Downing . 2007. “Using Multi‐Voxel Pattern Analysis of fMRI Data to Interpret Overlapping Functional Activations.” Trends in Cognitive Sciences 11, no. 1: 4–5. 10.1016/j.tics.2006.10.009. 17129747

[hbm70446-bib-0113] Peng, P. , J. Namkung , M. Barnes , and C. Sun . 2016. “A Meta‐Analysis of Mathematics and Working Memory: Moderating Effects of Working Memory Domain, Type of Mathematics Skill, and Sample Characteristics.” Journal of Educational Psychology 108, no. 4: 455–473. 10.1037/edu0000079.

[hbm70446-bib-0114] Peters, L. , and B. De Smedt . 2017. “Arithmetic in the Developing Brain: A Review of Brain Imaging Studies.” Developmental Cognitive Neuroscience 30: 265–279. 10.1016/j.dcn.2017.05.002. 28566139 PMC6969129

[hbm70446-bib-0115] Peterson, R. L. , R. Boada , L. M. McGrath , E. G. Willcutt , R. K. Olson , and B. F. Pennington . 2017. “Cognitive Prediction of Reading, Math, and Attention: Shared and Unique Influences.” Journal of Learning Disabilities 50, no. 4: 408–421. 10.1177/0022219415618500. 26825667 PMC4967036

[hbm70446-bib-0116] Pinheiro‐Chagas, P. , A. Daitch , J. Parvizi , and S. Dehaene . 2018. “Brain Mechanisms of Arithmetic: A Crucial Role for Ventral Temporal Cortex.” Journal of Cognitive Neuroscience 30, no. 12: 1757–1772. 10.1162/jocn_a_01319. 30063177 PMC6355388

[hbm70446-bib-0117] Pollack, C. , and N. C. Ashby . 2018. “Where Arithmetic and Phonology Meet: The Meta‐Analytic Convergence of Arithmetic and Phonological Processing in the Brain.” Developmental Cognitive Neuroscience 30: 251–264. 10.1016/j.dcn.2017.05.003. 28533112 PMC6969128

[hbm70446-bib-0118] Polspoel, B. , L. Peters , M. Vandermosten , and B. De Smedt . 2017. “Strategy Over Operation: Neural Activation in Subtraction and Multiplication During Fact Retrieval and Procedural Strategy Use in Children.” Human Brain Mapping 38, no. 9: 4657–4670. 10.1002/hbm.23691. 28626967 PMC6866817

[hbm70446-bib-0119] Power, J. D. , and S. E. Petersen . 2013. “Control‐Related Systems in the Human Brain.” Current Opinion in Neurobiology 23, no. 2: 223–228. 10.1016/j.conb.2012.12.009. 23347645 PMC3632325

[hbm70446-bib-0120] Prado, J. , R. Mutreja , H. Zhang , et al. 2011. “Distinct Representations of Subtraction and Multiplication in the Neural Systems for Numerosity and Language.” Human Brain Mapping 32: 1932–1947. 10.1002/hbm.21159. 21246667 PMC3117906

[hbm70446-bib-0121] Price, C. J. 2012. “A Review and Synthesis of the First 20years of PET and fMRI Studies of Heard Speech, Spoken Language and Reading.” NeuroImage 62, no. 2: 816–847. 10.1016/j.neuroimage.2012.04.062. 22584224 PMC3398395

[hbm70446-bib-0122] Price, G. R. , and D. Ansari . 2011. “Symbol Processing in the Left Angular Gyrus: Evidence From Passive Perception of Digits.” NeuroImage 57, no. 3: 1205–1211. 10.1016/j.neuroimage.2011.05.035. 21620978

[hbm70446-bib-0123] Purpura, D. J. , L. E. Hume , D. M. Sims , and C. J. Lonigan . 2011. “Early Literacy and Early Numeracy: The Value of Including Early Literacy Skills in the Prediction of Numeracy Development.” Journal of Experimental Child Psychology 110, no. 4: 647–658. 10.1016/j.jecp.2011.07.004. 21831396

[hbm70446-bib-0124] Ritchie, J. B. , S. Bracci , and H. Op de Beeck . 2017. “Avoiding Illusory Effects in Representational Similarity Analysis: What (Not) to Do With the Diagonal.” NeuroImage 148: 197–200. 10.1016/j.neuroimage.2016.12.079. 28069538

[hbm70446-bib-0125] Ritchie, J. B. , H. Lee Masson , S. Bracci , and H. P. Op de Beeck . 2021. “The Unreliable Influence of Multivariate Noise Normalization on the Reliability of Neural Dissimilarity.” NeuroImage 245: 118686. 10.1016/j.neuroimage.2021.118686. 34728244

[hbm70446-bib-0126] Ritchie, S. J. , and T. C. Bates . 2013. “Enduring Links From Childhood Mathematics and Reading Achievement to Adult Socioeconomic Status.” Psychological Science 24, no. 7: 1301–1308. 10.1177/0956797612466268. 23640065

[hbm70446-bib-0127] Rivera, S. M. , A. L. Reiss , M. A. Eckert , and V. Menon . 2005. “Developmental Changes in Mental Arithmetic: Evidence for Increased Functional Specialization in the Left Inferior Parietal Cortex.” Cerebral Cortex 15, no. 11: 1779–1790. 10.1093/cercor/bhi055. 15716474

[hbm70446-bib-0128] Rueckl, J. G. , P. M. Paz‐Alonso , P. J. Molfese , et al. 2015. “Universal Brain Signature of Proficient Reading: Evidence From Four Contrasting Languages.” Proceedings of the National Academy of Sciences 112, no. 50: 15510–15515. 10.1073/pnas.1509321112. PMC4687557 26621710

[hbm70446-bib-0129] Saban, W. , P. Pinheiro‐Chagas , S. Borra , and R. B. Ivry . 2024. “Distinct Contributions of the Cerebellum and Basal Ganglia to Arithmetic Procedures.” Journal of Neuroscience 44, no. 2: e1482222023. 10.1523/JNEUROSCI.1482-22.2023. 37973376 PMC10866191

[hbm70446-bib-0130] Scarborough, H. S. , and S. A. Brady . 2002. “Toward a Common Terminology for Talking About Speech and Reading: A Glossary of the “Phon” Words and Some Related Terms.” Journal of Literacy Research 34, no. 3: 299–336. 10.1207/s15548430jlr3403_3.

[hbm70446-bib-0131] Schlaggar, B. L. , and J. A. Church . 2009. “Functional Neuroimaging Insights Into the Development of Skilled Reading.” Current Directions in Psychological Science 18, no. 1: 21–26. 10.1111/j.1467-8721.2009.01599.x. 19750204 PMC2741313

[hbm70446-bib-0132] Schmahmann, J. D. 2019. “The Cerebellum and Cognition.” Neuroscience Letters 688: 62–75. 10.1016/j.neulet.2018.07.005. 29997061

[hbm70446-bib-0133] Seidenberg, M. S. , and J. L. McClelland . 1989. “A Distributed, Developmental Model of Word Recognition and Naming.” Psychological Review 96, no. 4: 523–568. 10.1037/0033-295X.96.4.523. 2798649

[hbm70446-bib-0134] Shum, J. , D. Hermes , B. L. Foster , et al. 2013. “A Brain Area for Visual Numerals.” Journal of Neuroscience 33, no. 16: 6709–6715. 10.1523/JNEUROSCI.4558-12.2013. 23595729 PMC3970733

[hbm70446-bib-0135] Šidák, Z. 1967. “Rectangular Confidence Regions for the Means of Multivariate Normal Distributions.” Journal of the American Statistical Association 62, no. 318: 626–633. 10.1080/01621459.1967.10482935.

[hbm70446-bib-0136] Siegler, R. S. 1987. “The Perils of Averaging Data Over Strategies: An Example From Children's Addition.” Journal of Experimental Psychology: General 116, no. 3: 250–264. 10.1037/0096-3445.116.3.250.

[hbm70446-bib-0137] Siegler, R. S. 1988. “Strategy Choice Procedures and the Development of Multiplication Skill.” Journal of Experimental Psychology: General 117, no. 3: 258–275. 10.1037/0096-3445.117.3.258. 2971762

[hbm70446-bib-0138] Simmons, F. R. , and C. Singleton . 2008. “Do Weak Phonological Representations Impact on Arithmetic Development? A Review of Research Into Arithmetic and Dyslexia.” Dyslexia 14, no. 2: 77–94. 10.1002/dys.341. 17659647

[hbm70446-bib-0139] Simon, O. , J.‐F. Mangin , L. Cohen , D. L. Bihan , and S. Dehaene . 2002. “Topographical Layout of Hand, Eye, Calculation, and Language‐Related Areas in the Human Parietal Lobe.” Neuron 33, no. 3: 475–487. 10.1016/S0896-6273(02)00575-5. 11832233

[hbm70446-bib-0164] Singer, V. , and K. Strasser . 2017. “The Association Between Arithmetic and Reading Performance in School: A Meta‐Analytic Study.” School Psychology Quarterly 32, no. 4: 435–448. 10.1037/spq0000197. 28221052

[hbm70446-bib-0140] Slot, E. M. , S. van Viersen , E. H. de Bree , and E. H. Kroesbergen . 2016. “Shared and Unique Risk Factors Underlying Mathematical Disability and Reading and Spelling Disability.” Frontiers in Psychology 7: 803. 10.3389/fpsyg.2016.00803. 27375508 PMC4901067

[hbm70446-bib-0141] Snowling, M. J. , K. Moll , and C. Hulme . 2021. “Language Difficulties Are a Shared Risk Factor for Both Reading Disorder and Mathematics Disorder.” Journal of Experimental Child Psychology 202: 105009. 10.1016/j.jecp.2020.105009. 33126134 PMC7677889

[hbm70446-bib-0142] Spiegel, J. A. , J. M. Goodrich , B. M. Morris , C. M. Osborne , and C. J. Lonigan . 2021. “Relations Between Executive Functions and Academic Outcomes in Elementary School Children: A Meta‐Analysis.” Psychological Bulletin 147: 329–351. 10.1037/bul0000322. 34166004 PMC8238326

[hbm70446-bib-0143] Stanescu‐Cosson, R. , P. Pinel , P.‐F. Van De Moortele , D. Le Bihan , L. Cohen , and S. Dehaene . 2000. “Understanding Dissociations in Dyscalculia.” Brain 123, no. 11: 2240–2255. 10.1093/brain/123.11.2240. 11050024

[hbm70446-bib-0144] Steiger, J. H. 1980. “Tests for Comparing Elements of a Correlation Matrix.” Psychological Bulletin 87, no. 2: 245–251. 10.1037/0033-2909.87.2.245.

[hbm70446-bib-0145] Stiers, P. , M. Mennes , and S. Sunaert . 2010. “Distributed Task Coding Throughout the Multiple Demand Network of the Human Frontal–Insular Cortex.” NeuroImage 52, no. 1: 252–262. 10.1016/j.neuroimage.2010.03.078. 20362676

[hbm70446-bib-0146] Thompson, R. , and J. Duncan . 2009. “Attentional Modulation of Stimulus Representation in Human Fronto‐Parietal Cortex.” NeuroImage 48, no. 2: 436–448. 10.1016/j.neuroimage.2009.06.066. 19577650

[hbm70446-bib-0147] Torgesen, J. K. , R. K. Wagner , and C. A. Rashotte . 1994. “Longitudinal Studies of Phonological Processing and Reading.” Journal of Learning Disabilities 27, no. 5: 276–286. 10.1177/002221949402700503. 8006506

[hbm70446-bib-0148] Trbovich, P. L. , and J.‐A. LeFevre . 2003. “Phonological and Visual Working Memory in Mental Addition.” Memory & Cognition 31, no. 5: 738–745. 10.3758/BF03196112. 12956238

[hbm70446-bib-0149] Uittenhove, K. , C. Thevenot , and P. Barrouillet . 2016. “Fast Automated Counting Procedures in Addition Problem Solving: When Are They Used and Why Are They Mistaken for Retrieval?” Cognition 146: 289–303. 10.1016/j.cognition.2015.10.008. 26491834

[hbm70446-bib-0150] Ünal, Z. E. , N. R. Greene , X. Lin , and D. C. Geary . 2023. “What Is the Source of the Correlation Between Reading and Mathematics Achievement? Two Meta‐Analytic Studies.” Educational Psychology Review 35, no. 1: 4. 10.1007/s10648-023-09717-5.

[hbm70446-bib-0151] Vanbinst, K. , E. van Bergen , P. Ghesquière , and B. De Smedt . 2020. “Cross‐Domain Associations of Key Cognitive Correlates of Early Reading and Early Arithmetic in 5‐Year‐Olds.” Early Childhood Research Quarterly 51: 144–152. 10.1016/j.ecresq.2019.10.009.

[hbm70446-bib-0152] Vogel, S. E. , and B. De Smedt . 2021. “Developmental Brain Dynamics of Numerical and Arithmetic Abilities.” npj Science of Learning 6, no. 1: 22. 10.1038/s41539-021-00099-3. 34301948 PMC8302738

[hbm70446-bib-0153] Vukovic, R. K. , and N. K. Lesaux . 2013. “The Relationship Between Linguistic Skills and Arithmetic Knowledge.” Learning and Individual Differences 23: 87–91. 10.1016/j.lindif.2012.10.007.

[hbm70446-bib-0154] Wagner, R. K. , and J. K. Torgesen . 1987. “The Nature of Phonological Processing and Its Causal Role in the Acquisition of Reading Skills.” Psychological Bulletin 101: 192–212.

[hbm70446-bib-0155] Walther, A. , H. Nili , N. Ejaz , A. Alink , N. Kriegeskorte , and J. Diedrichsen . 2016. “Reliability of Dissimilarity Measures for Multi‐Voxel Pattern Analysis.” NeuroImage 137: 188–200. 10.1016/j.neuroimage.2015.12.012. 26707889

[hbm70446-bib-0156] Whitfield‐Gabrieli, S. , and A. Nieto‐Castanon . 2012. “Conn: A Functional Connectivity Toolbox for Correlated and Anticorrelated Brain Networks.” Brain Connectivity 2, no. 3: 125–141. 10.1089/brain.2012.0073. 22642651

[hbm70446-bib-0157] Willcutt, E. G. , L. M. McGrath , B. F. Pennington , et al. 2019. “Understanding Comorbidity Between Specific Learning Disabilities.” New Directions for Child and Adolescent Development 2019, no. 165: 91–109. 10.1002/cad.20291. PMC6686661 31070302

[hbm70446-bib-0158] Wong, T. T.‐Y. , C. S.‐H. Ho , and J. Tang . 2016. “The Relation Between ANS and Symbolic Arithmetic Skills: The Mediating Role of Number‐Numerosity Mappings.” Contemporary Educational Psychology 46: 208–217. 10.1016/j.cedpsych.2016.06.003.

[hbm70446-bib-0159] Woolgar, A. , R. Thompson , D. Bor , and J. Duncan . 2011. “Multi‐Voxel Coding of Stimuli, Rules, and Responses in Human Frontoparietal Cortex.” NeuroImage 56, no. 2: 744–752. 10.1016/j.neuroimage.2010.04.035. 20406690

[hbm70446-bib-0160] Yeo, D. J. , E. D. Wilkey , and G. R. Price . 2017. “The Search for the Number Form Area: A Functional Neuroimaging Meta‐Analysis.” Neuroscience & Biobehavioral Reviews 78: 145–160. 10.1016/j.neubiorev.2017.04.027. 28467892

[hbm70446-bib-0161] Zbrodoff, N. J. , and G. D. Logan . 2005. “What Everyone Finds: The Problem‐Size Effect.” In Handbook of Mathematical Cognition, 331–345. Psychology Press. 10.4324/9780203998045.

