# Supplementary material for: Crossroads in the Learning Brain: The Neural Overlap Between Arithmetic and Phonological Processing
Source: Hum Brain Mapp. 2026 Jan 13;47(1):e70446. doi: 10.1002/hbm.70446 (PMC12797254; doi:10.1002/hbm.70446)
Supplement: Supplementary file 1 — Data S1: hbm70446‐sup‐0001‐Supinfo.docx. [file HBM-47-e70446-s001.docx]

1. **Description of the similarity analysis including multivariate noise normalization and split-half correlations.**

In order to test if each individual similarity value was significant, we split the data into two halves corresponding to different experimental runs. Patterns for each condition (Small, Large, Rhyming) were estimated for each run separately. Since the current dataset includes two runs for each task, this resulted in two separate, run-specific patterns for each condition (6 patterns in total). For any given pair of conditions (Rhyming and Small; or Rhyming and Large), new similarity estimates were obtained by calculating the correlation between each of the patterns for one condition with each of the patterns for the other, resulting in 4 separate estimates (2 Conditions x 2 Runs) that were then averaged to obtain the final similarity value. We tested if the cross-validated correlations were significantly different from 0 using a t-test. Significance was initially assessed using an uncorrected alpha of 0.05, which was then corrected for multiple comparisons using Dun-Šidák corrections.

Throughout this split-half procedure, we also employed multivariate noise normalization for the estimation of the patterns (Diedrichsen et al., 2011; Kriegeskorte et al., 2008). This procedure is implemented to account for the effects of spatially correlated noise that is usually observed across neighboring voxels in fMRI data without changing the structure of correlations between patterns. To implement this normalization, we built a noise covariance matrix using the residuals from the first-level GLM and estimated what transformation needs to be applied in order to make the noise terms for each voxel uncorrelated. Then, this transformation is applied to the data to remove any effects of spatially correlated noise from the patterns that will be used for the analysis. This procedure was performed on each participant. According to previous literature, inferences about the relationships among noise-normalized patterns show increased reliability (Walther et al., 2016; Misaki et al., 2010; Guggenmos et al., 2018).

In summary, multivariate noise normalization, on the one hand, can mitigate the effects of spatially correlated noise, which would result in more reliable patterns (although its effectiveness is currently under debate; see Charest et al., 2018; Liu et al., 2021; Ritchie et al., 2021) while the use of split-half correlations can reduce the effect of temporal autocorrelation between patterns that are acquired closer in time by only considering correlations between patterns obtained in different runs. Therefore, we expect the current analysis to further robustness to the statistical inferences about the relationships between different experimental conditions already planned in the original preregistration.

1. **Brain activation associated to arithmetic and phonological processing in adults**

| **Small + Large Problems > Plus One** | | | |
| --- | --- | --- | --- |
| Cluster | Size | Peak coordinate | Description |
| 1  2  3  4  5  6  7  8  9  10  11  12  13 | 3354  817  663  596  268  167  52  36  29  28  28  25  18 | [35, -60, -38]  [-26, 24, -13]  [-29, -75, 24]  [30, 24, -10]  [15, 29,24]  [35, -42, 29]  [-51, 4, 44]  [0, -20, -18]  [-27, -25, -8]  [-22, -58, 0]  [15, -11, 4]  [-14, -10, 2]  [5, -30, -5] | Bilateral visual association areas and posterior cerebellum  Left inferior frontal and premotor cortex  Left parieto-occipital cortex  Right ventrolateral prefrontal cortex and anterior insula  Bilateral medial frontal cortex and supplementary motor area  Right parieto-occipital cortex  Left precentral gyrus  Right midbrain  Left thalamus and hippocampus  Left precuneus  Right thalamus  Left thalamus  Right midbrain |
| **Rhyming > Letter Matching** | | | |
| Cluster | Size | Peak coordinate | Description |
| 1  2  3  4  5  6  7  8  9  10  11  12  13  14  15 | 1528  296  879  161  143  120  80  59  57  32  29  28  25  20  18 | [-54, 21, 22]  [-4, 44, 44]  [-63, -48, -8]  [42, -72, -35]  [-29, -15, -15]  [-7, 51, -18]  [35, 34, -13]  [-39, 67, 34]  [-44, 12, 47]  [57, -5, -8]  [20, -8, -18]  [-9, -57, 32]  [-4, -55, 17]  [-4, -35, 34]  [-41, -42, -18] | Left ventrolateral and orbitofrontal cortex  Left medial-frontal cortex and supplementary motor area  Left inferior, middle and superior temporal cortex  Right posterior cerebellum  Left hippocampus  Left medial orbitofrontal cortex  Right orbitofrontal cortex  Left angular gyrus  Left middle frontal cortex  Right middle and superior temporal cortex  Right hippocampus  Left precuneus  Left precuneus  Left middle and posterior cingulate gyrus  Left inferior temporal cortex |

1. **Brain activation associated to arithmetic and phonological processing in Children**

| **Small + Large Problems > Plus One** | | | |
| --- | --- | --- | --- |
| Cluster | size | Peak coordinate | Anatomical location of the Peak Coordinate |
| 1  2  3  4  5  6  7  8  9  10  11  12  13  14  15 | 4723  577  432  369  279  196  189  178  138  99  98  92  61  53  44 | [-17, -97 ,0]  [33, 53, 42]  [13, 24, 39]  [-46, 6, 32]  [30, 2, 54]  [30, 24, 0]  [-32, 24, 0]  [-29, 6, 59]  [-51, -60, -5]  [28, -65, -28]  [-6, -28, -8]  [43, 36, 17]  [50, 11, 32]  [-46, 48, 2]  [-51, 39, 17] | Bilateral occipito-parietal cortex and cerebellum  Right parieto-occipital cortex  Bilateral supplemental motor area and medial frontal cortex  Left inferior frontal and precentral cortex  Right middle and superior frontal cortex  Right insular and inferior frontal cortex  Left insular and inferior frontal cortex  Left middle frontal and precentral cortex  Left inferior and middle temporal cortex  Right posterior cerebellum  Midbrain  Right middle and inferior frontal cortex  Right inferior frontal and precentral cortex  Left inferior and middle frontal gyrus  Left inferior frontal gyrus |
| **Rhyming > Letter Matching** | | | |
| Cluster | size | Peak coordinate | Anatomical location of the Peak Coordinate |
| 1  2  3  4 | 3255  176  153  85 | [-54, 33, 14]  [-7, 16, 59]  [-44, -23, -20]  [-22, -15, -15] | Left fronto-temporal cortex  Bilateral supplemental motor area and superior frontal cortex  Left fusiform and parahippocampal gyri  Left hippocampus |

1. **Children vs Adults**

The conjunction analyses conducted separately for each group revealed different regions of overlap between the two tasks in children and adults. Even within the inferior frontal gyrus—the only anatomical region showing significant conjunction effects in both groups—the specific voxels involved differed substantially (Figure S3). In adults, the overlap was located almost exclusively in the triangular part of the inferior frontal gyrus. In contrast, children showed two distinct clusters in this region: one situated more anteriorly within the triangular section (IFG2), and another more posterior cluster extending from the triangular into the opercular section.


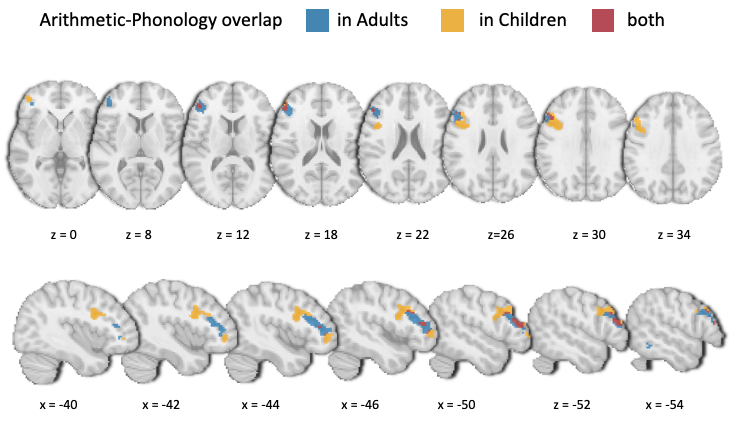


*Figure S1. Overlap between arithmetic and reading along the inferior frontal cortex for Adults and Children*

One possibility is that differences in overlapping regions are due to fundamental differences in the areas of the brain recruited during each task by children vs adults. However, direct comparison between the groups across each condition of interest (Small, Large, Rhyming) indicated very minimal differences. For Small problems, differences between groups were found in a cluster on the precuneus (Figure S2), where significant deactivation relative to the control was observed in the Adults but not the Children. For Large problems, group differences were observed on the fusiform gyrus, the medial frontal cortex and the posterior cingulate cortex. Figure 2.6B shows that group differences in these areas arise from a high level of deactivation in the Children group along these areas during Large problems compared to the control condition. No significant differences were observed between the groups for the brain activation corresponding to the rhyming task.


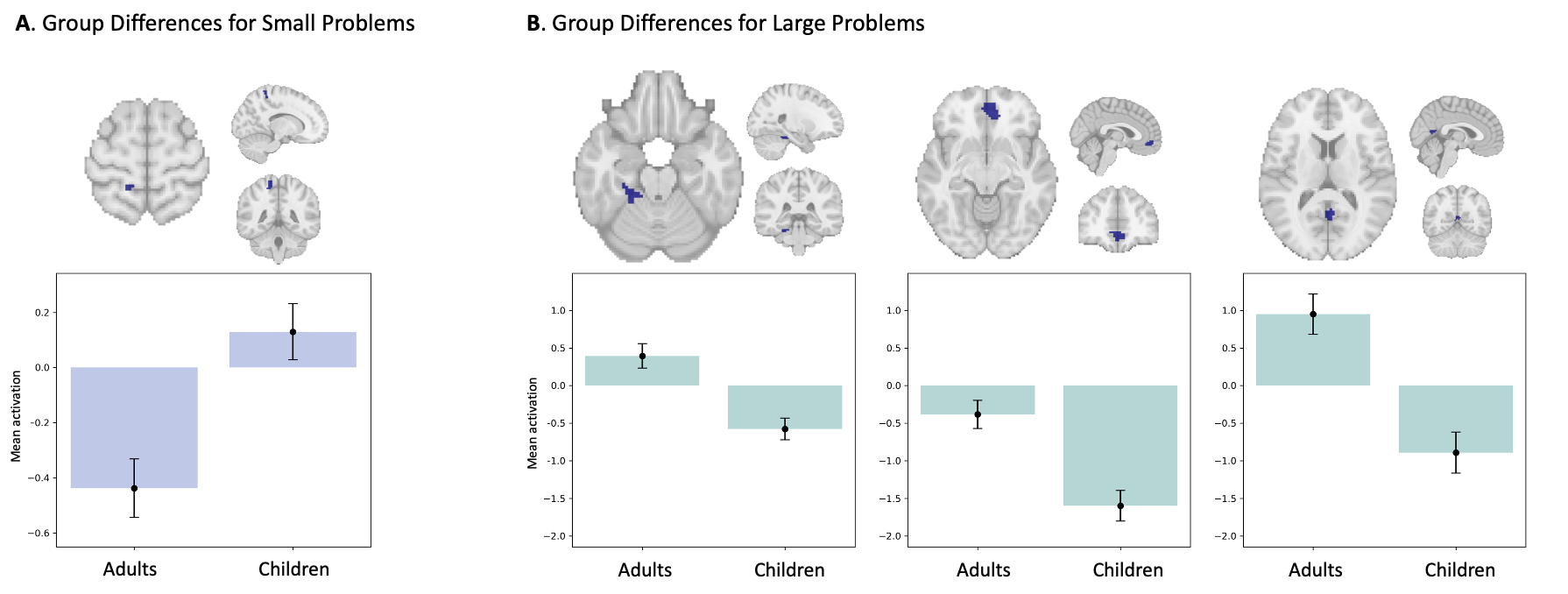


*Figure S2. Group Differences for Small and Large Problems*

**References**

Charest, I., Kriegeskorte, N., & Kay, K. N. (2018). GLMdenoise improves multivariate pattern analysis of fMRI data. *NeuroImage*, *183*, 606–616. https://doi.org/10.1016/j.neuroimage.2018.08.064

Diedrichsen, J., Ridgway, G. R., Friston, K. J., & Wiestler, T. (2011). Comparing the similarity and spatial structure of neural representations: A pattern-component model. *NeuroImage*, *55*(4), 1665–1678. https://doi.org/10.1016/j.neuroimage.2011.01.044

Guggenmos, M., Sterzer, P., & Cichy, R. M. (2018). Multivariate pattern analysis for MEG: A comparison of dissimilarity measures. *NeuroImage*, *173*, 434–447. https://doi.org/10.1016/j.neuroimage.2018.02.044

Kriegeskorte, N., Mur, M., & Bandettini, P. (2008). Representational similarity analysis—Connecting the branches of systems neuroscience. *Frontiers in Systems Neuroscience*, *2*. https://www.frontiersin.org/article/10.3389/neuro.06.004.2008

Liu, P., Chrysidou, A., Doehler, J., Hebart, M. N., Wolbers, T., & Kuehn, E. (2021, May 18). *The organizational principles of de-differentiated topographic maps in somatosensory cortex*. eLife; eLife Sciences Publications Limited. https://doi.org/10.7554/eLife.60090

Misaki, M., Kim, Y., Bandettini, P. A., & Kriegeskorte, N. (2010). Comparison of multivariate classifiers and response normalizations for pattern-information fMRI. *NeuroImage*, *53*(1), 103–118. https://doi.org/10.1016/j.neuroimage.2010.05.051

Ritchie, J. B., Lee Masson, H., Bracci, S., & Op de Beeck, H. P. (2021). The unreliable influence of multivariate noise normalization on the reliability of neural dissimilarity. *NeuroImage*, *245*, 118686. https://doi.org/10.1016/j.neuroimage.2021.118686

Walther, A., Nili, H., Ejaz, N., Alink, A., Kriegeskorte, N., & Diedrichsen, J. (2016). Reliability of dissimilarity measures for multi-voxel pattern analysis. *NeuroImage*, *137*, 188–200. https://doi.org/10.1016/j.neuroimage.2015.12.012
